# FTO degrader impairs ribosome biogenesis and protein translation in acute myeloid leukemia

**DOI:** 10.1126/sciadv.adv7648

**Published:** 2025-08-15

**Authors:** Wenlong Li, Yutao Zhao, Dong Wu, Zhenhua Chen, Ying Qing, Fan Yang, Fei Ji, Linda Zhang, Lillian Sau, Jianjun Chen, Chuan He

**Affiliations:** ^1^ Departments of Chemistry, Department of Biochemistry and Molecular Biology, Institute for Biophysical Dynamics, University of Chicago, Chicago, IL 60637, USA.; ^2^ Howard Hughes Medical Institute, University of Chicago, Chicago, IL 60637, USA.; ^3^ Department of Systems Biology, Beckman Research Institute of City of Hope, Duarte, CA 91010, USA.; ^4^ Center for RNA Biology and Therapeutics, Beckman Research Institute of City of Hope, Duarte, CA 91010, USA.; ^5^ Department of Hematology, The First Affiliated Hospital, Zhejiang Provincial Key Laboratory of Hematopoietic Malignancy, School of Medicine, Zhejiang University, Hangzhou, 310058, China.; ^6^ Department of Radiation and Cellular Oncology, The University of Chicago, Chicago, IL 60637, USA.; ^7^ Ludwig Center for Metastasis Research, The University of Chicago, Chicago, IL 60637, USA.

## Abstract

Targeting ribosome biogenesis and protein translation has emerged as a promising avenue for cancer therapy. The fat mass and obesity-associated protein (FTO), an RNA *N*^6^-methyladenosine (m^6^A) eraser, has been identified as an oncogenic factor in acute myeloid leukemia (AML). Here, we present the development of an FTO degrader that selectively degrades FTO in AML cells, demonstrating superior efficacy both in vitro and in vivo. We confirmed that FTO degradation increases m^6^A modifications on mRNAs associated with ribosome biogenesis, promoting their YTHDF2-mediated decay. This disruption of ribosome biogenesis and protein translation contributes to the inhibition of AML progression. Our findings highlight this FTO degrader as a valuable tool compound for elucidating the functional roles of FTO in cancer and as a potential foundation for the development of selective anticancer therapies.

## Teaser

FTO degrader impairs ribosome biogenesis in acute myeloid leukemia.

## INTRODUCTION

*N*^6^-methyladenosine (m^6^A) is the most prevalent internal modification of eukaryotic mRNAs, playing critical roles in multiple cellular biological functions, including RNA processing, stability, translation, subcellular localization and RNA structural regulation (*1*). The m^6^A modification is mainly installed by the writer complex, comprising METTL3 and METTL14 (*2*), and erased by the demethylases ALKBH5 and fat mass and obesity-associated protein (FTO) (*3*, *4*). Accumulating evidence suggests that the intricate regulatory network of this reversible modification is profoundly associated with human diseases (*5*). Dysregulated m^6^A effectors have been shown to function as either oncoproteins or tumor suppressors, playing important roles in cancer initiation, progression, and metastasis (*1*, *6*, *7*).

Acute myeloid leukemia (AML) is a malignant disorder of myeloid hematopoietic stem and progenitor cells (*8*). Emerging evidences suggest that m^6^A modification plays a crucial role in AML development (*9*). Key m^6^A regulators, including the writer METTL3 and its binding partner METTL14, as well as the eraser FTO, have been identified as promising therapeutic targets for AML treatment (*10*, *11*). The notable therapeutic efficacy of small molecules targeting FTO, such as FB23-2 (*12*), FTO degrader QP73 (*13*), CS-1, and CS-2 (*14*), further validated the potential of targeting FTO in antileukemia therapies.

Mechanistically, FTO removes m^6^A modifications from mRNA targets, such as *SOCS box protein 2* and *retinoic acid receptor* α, triggering signaling cascades that inhibit myeloid differentiation and promote leukemogenesis (*11*). In addition, FTO enhances the stability of *MYC* and *CEBPA* mRNAs by erasing their m^6^A marks, thereby facilitating their oncogenic roles (*15*). FTO also regulates *FOXO3* expression, contributing to chemotherapy resistance in AML cells (*16*).

Translation depends on the complex and tightly regulated process of ribosome biogenesis, with ribosomes serving as the effectors of translation through their critical roles in mRNA decoding and protein synthesis. (*17*, *18*). Dysregulated ribosome biogenesis has been strongly linked to tumorigenesis, as it supports the high translational demands of rapidly proliferating cancer cells (*19*). Recent studies demonstrated the crucial roles that ribosome biogenesis plays in the development of AML, indicating it as a potential therapeutic strategy (*20*–*22*). In this study, we describe the development of an FTO degrader that selectively degrades FTO in AML cells, demonstrating superior efficacy both in vitro and in vivo. Using this tool compound, we further uncovered a regulatory pathway by which FTO degradation increases m^6^A modifications on ribosome biogenesis–related mRNAs, promoting their YTHDF2-mediated degradation. This process disrupts ribosome biogenesis and impairs global translation, particularly the translation of DNA replication–related genes, contributing to AML progression inhibition.

## RESULTS

### Design and optimization of FTO-targeted PROTAC degrader

**Fig. 1.**
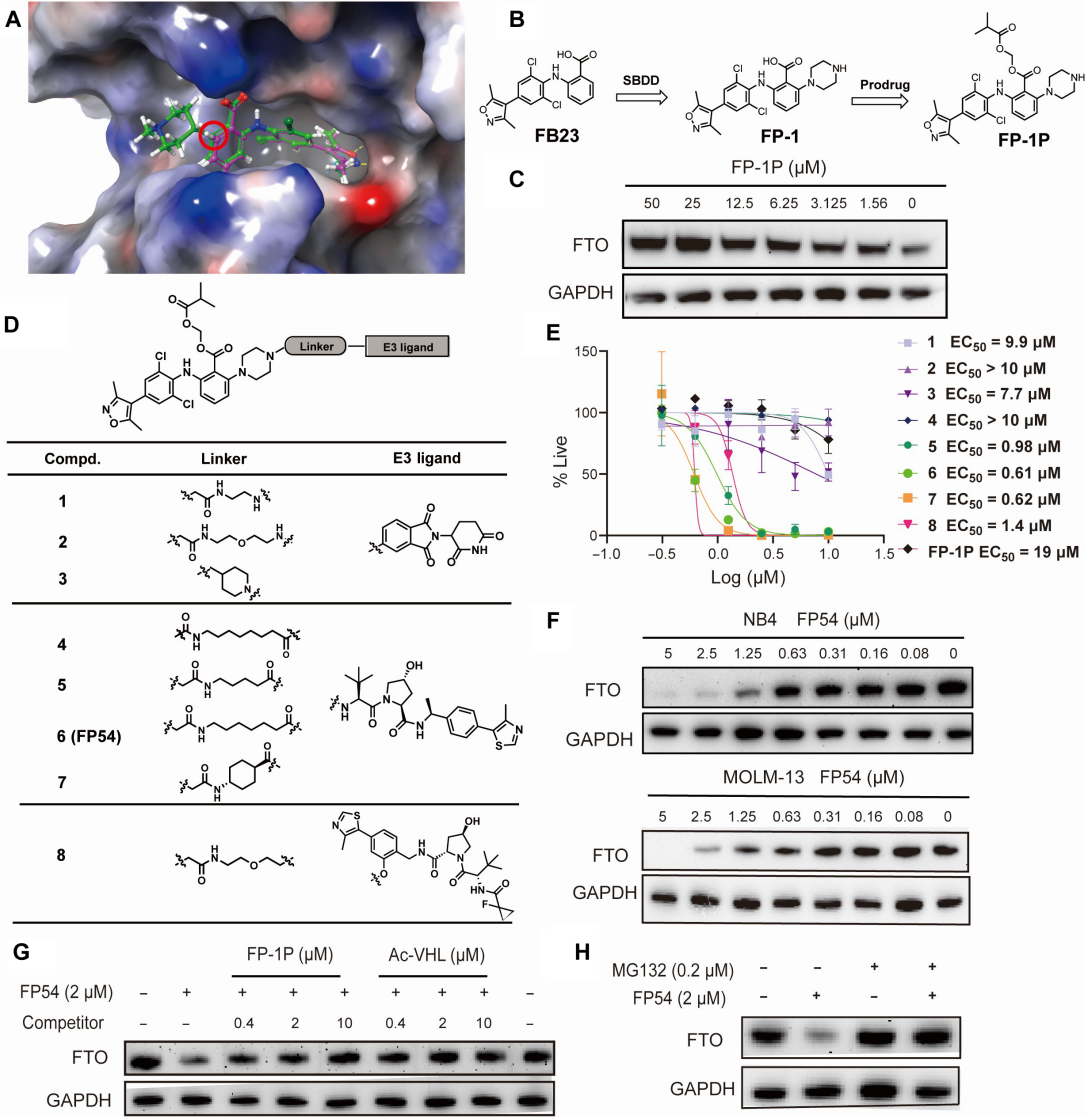
FP54 induces degradation of FTO through the ubiquitin-proteasomal system. (**A**) Molecule modeling revealed a high degree of overlap between compound FP-1 (green) and FB23 (magenta) within the FTO binding pocket (PDB: 6AKW). (**B**) Design strategies of prodrug form FP-1P. (**C**) CETSA to examine interaction of compound FP-1P at a series of concentrations with FTO. (**D**) Chemical structures of synthesized FTO degraders **1** to **8**. (**E**) Antiproliferative activities of compound **1** to **8** and FP-1P in NB4 cells. Values in the plot are shown as mean ± SD from *n* = 3 independent experiments. (**F**) Immunoblots for FTO and glyceraldehyde phosphate dehydrogenase (GAPDH) in NB4 and MOLM-13 cells after 48-hour treatment of FP54 at different concentrations. (**G**) Immunoblots for FTO and GAPDH in NB4 cells pretreated with the indicated concentration of FP-1P and Ac-VHL for 2 hours, followed by a 48-hour treatment with 2 μM FP54. (**H**) Immunoblots for FTO and GAPDH in NB4 cells pretreated with the indicated concentration of MG132 for 2 hours, followed by a 48-hour treatment with 2 μM FP54. Data shown in (C) and (F) to (H) are representative of three independent experiments. EC_50_, median effective concentration.

We selected FB23 as the FTO binder part for our proteolysis-targeting chimera (PROTAC) design as it is a well-characterized FTO inhibitor, although its potency in cellular assays was low (*12*). On the basis of the binding mode of FB23 with FTO (Fig. 1A), we observed that the C-6 position on the phenyl ring, which is adjacent to the carboxyl group, is oriented toward the solvent-exposed direction. This structural insight informed our decision to synthesize FP-1 (Fig. 1B) by introducing a piperazine to serve as the anchor point for subsequent PROTAC design. Molecular docking results showed that FP-1 overlapped very well with FB23 when binding to FTO (Fig. 1A). Recognizing that the carboxyl group of FB23 is critical for its FTO inhibitory activity but contributes to poor cellular permeability, we opted to enhance cell entry of FP-1 by leveraging an ester prodrug approach. Consequently, compound FP-1P (Fig. 1B) was synthesized. Cellular thermal shift assay (CETSA) confirmed its direct interaction with FTO within NB4 cells, indicating dose-dependent binding with FTO (Fig. 1C). Cell proliferation assay demonstrated improved cellular activity of FP-1P when compared with FB23 (fig. S1, A and B). On the basis of the preliminary success of FP-1P, we designed and synthesized a series of heterobifunctional compounds (**1** to **8**), exploring various linkers and several ligands of E3 ligases, such as cesreblon (CRBN) and von Hippel-Lindau (VHL) (Fig. 1D). We evaluated their effects on degrading FTO (fig. S2, A to D).

Compounds **1** to **3**, having the CRBN ligand, exhibited only minor degradation activity at 10 μM. Compounds **5**, **6** (FP54), and **7**, containing the VHL ligand, all completely degraded FTO at 10 μM. The introduction of an amide linker in compound **4** led to no obvious degradation activity. In addition, we explored an alternative VHL-binding ligand that was present in compound **8** (*23*) and found that it could also induce FTO degradation at 10 μM, although its maximal degradation activity was less potent compared to those of compounds **6** to **8**. The antiproliferative activities of compounds **1** to **8** correlated well with their activities in inducing FTO degradation in NB4 cells (Fig. 1E and fig. S2, A to D). Among them, compounds **5** to **7** showed the most potent antiproliferative activities, surpassing the efficacy of the reported FTO inhibitor FB23-2, particularly in NOMO-1 and SKM-1 cells (fig. S1, C to F).

### FP54 effectively degrades FTO in AML cells

We further characterized the efficiency of FP54 on degrading FTO within leukemia cells. In NB4 and MOLM-13 cells, FP54 induced the degradation of FTO in a dose-dependent manner, with maximum degradation exceeding 95%. No hook effect was observed during the experiment (Fig. 1F). Compounds **5** and **7** also displayed similar degradation profiles (fig. S2, E to G). The degradation half-maximal concentration of FP54 was calculated to be 0.68 μM in NB4 cells and 0.71 μM in MOLM-13 cells with the *D*_max_ more than 95% (fig. S3A). FP54 did not induce degradation of other RNA m^6^A effector proteins, including YTHDF2, METTL3, and ALKBH5 in NB4 cells (fig. S3B). To validate that the FTO degradation activity of FP54 depends on VHL, we synthesized a negative control compound (FP54-NC) that lacks the ability to bind to VHL (fig. S3C). This compound did not show any degradation of FTO even at concentrations up to 10 μM (fig. S3D).

FP54 was more effective than FP54-NC in inhibiting the proliferation of NB4 and MOLM-13 cells (fig. S3E). Despite its inability to recruit VHL for targeted degradation, FP54-NC still exhibited improved activity compared to FP-1P, which may be attributed to enhanced cellular permeability facilitated by the presence of the VHL skeleton. In the presence of a VHL ligand, Ac-VHL (*24*), the antiproliferative activity of FP54 notably decreased (fig. S3F), suggesting that the cell activity of FP54 depends on the VHL. However, FP54 exhibited a slower degradation rate than the reported PROTACs (fig. S3G) (*24*, *25*), which could be attributed to the additional time required for the hydrolysis of the prodrug form. Pretreatment with either FP-1P or Ac-VHL (*24*) dose-dependently blocked the FP54-induced FTO degradation (Fig. 1G). In addition, pretreatment with MG132, a proteasome inhibitor, completely abolished the FP54-induced FTO degradation (Fig. 1H), confirming that the degradation process is ubiquitin-proteasome system dependent. Together, these results collectively confirm that FP54 induces FTO degradation primarily through a mechanism that relies on the recruitment of the VHL E3 ligase complex and subsequent ubiquitin-proteasome–mediated degradation.

### FP54 selectively inhibits proliferation and promotes apoptosis of AML cells

**Fig. 2.**
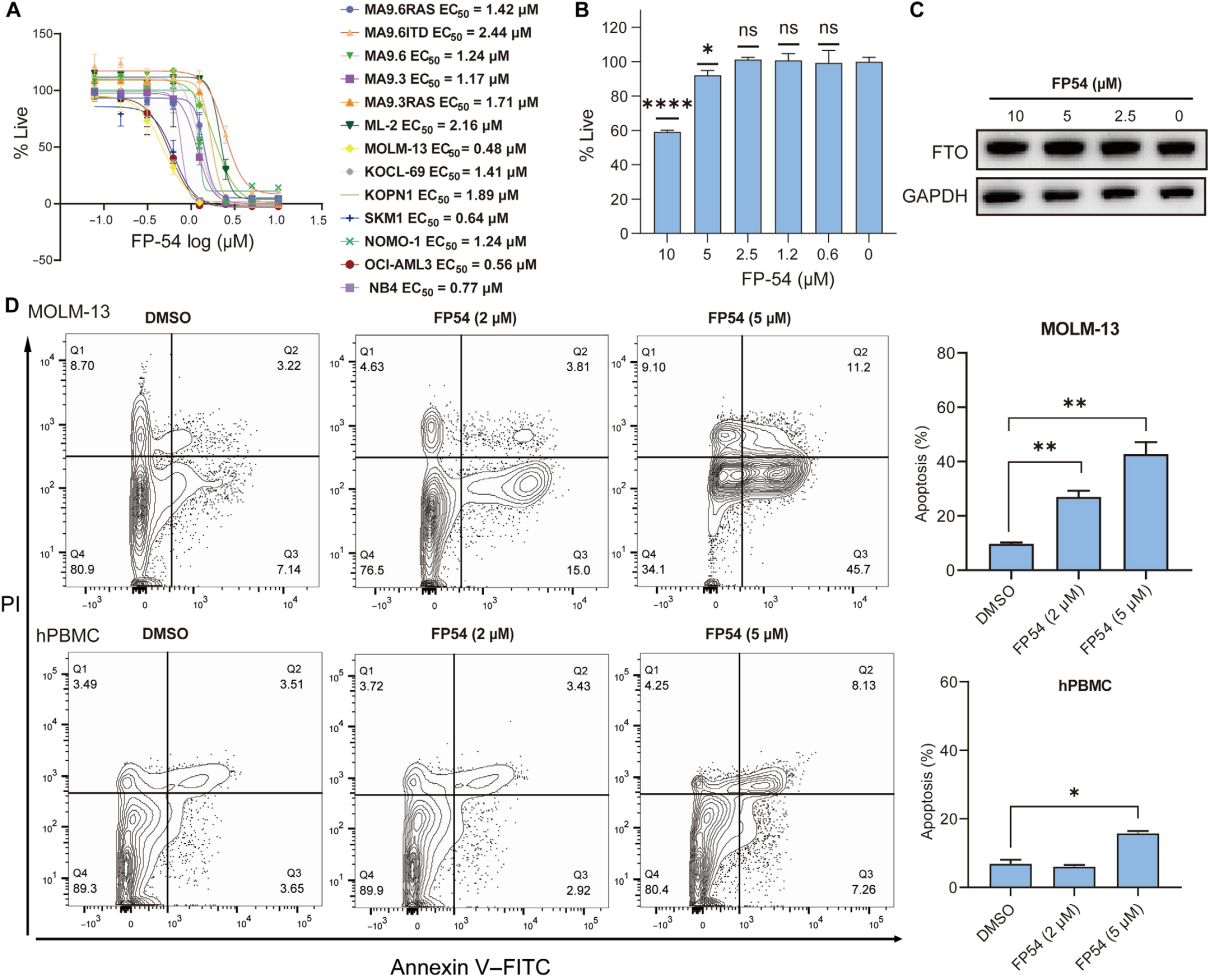
FP54 selectively inhibits the proliferation and induces the apoptosis of AML cells. (**A**) Antiproliferative activities of FP54 in a panel of AML cells. Values in the plot are shown as mean ± SD from *n* = 3 independent experiments. (**B**) Inhibitory effects of FP54 on hPBMCs after treatment for 72 hours. Data are reported as mean ± SD of *n* = 4 independent replicates. (**C**) Immunoblots for FTO and GAPDH in hPBMCs after treatment with indicated concentrations of FP54 for 48 hours. Blot is representative of three independent experiments. (**D**) Apoptosis quantification in MOLM-13 and hPBMCs treated with DMSO and FP54 at indicated concentrations for 48 hours (*n* = 3). The propidium iodide (PI)– and annexin V–positive cells were quantified by flow cytometry. *P < 0.05, **P < 0.01, and ****P < 0.0001; unpaired Student’s *t* test. ns, not significant.

We evaluated the antileukemia effects of FP54 across a diverse panel of AML cell lines, and FP54 efficiently inhibited the proliferation of these AML cell lines with median inhibitory concentration (IC_50_) values ranging from 0.48 to 2.44 μM, despite their varied genetic backgrounds (Fig. 2A), which is also confirmed by the colony formation assay in NB4 and MOLM-13 cells (fig. S4A). In contrast, FP54 demonstrated considerably less cytotoxicity in human peripheral blood mononuclear cells (hPBMCs), with toxicity beginning to emerge at 5 μM, likely due to its FTO inhibition activity (Fig. 2B). We attributed this reduced toxicity of FP54 in hPBMCs to its inability of degrading FTO in normal cells (Fig. 2C). This selectivity is likely due to the differential expression of VHL between tumor cells and normal tissues (*26*), allowing FP54 to target and degrade FTO more specifically in tumor cells while sparing normal cells. Similarly, FP54 significantly induced apoptosis in MOLM-13 (Fig. 2D) and NB4 cells (fig. S4E), whereas its effect on hPBMCs was notably weaker (Fig. 2D). These results suggest that FP54 has the potential to selectively target AML cells while sparing normal cells. To further confirm its proapoptotic effect in leukemia cells, we showed that FP54 induced the cleavage of caspase-3 and poly(ADP-ribose) polymerase 1 starting at 1 μM in both MOLM-13 and NB4 cells (fig. S4, B and C). In contrast, FP54 could trigger apoptosis in hPBMCs only at a higher concentration (5 μM), likely as a result of FTO inhibition (fig. S4D). Furthermore, FP54 demonstrated significant effects on the differentiation markers in leukemia cells, notably increasing both CD11b and CD14 expression at a concentration of 5 μM in NB4 cells; it also increased CD14 expression in MOLM-13 cells although not the CD11b expression (fig. S4F). Furthermore, liquid chromatography–mass spectrometry analysis revealed that the FP54 treatment significantly increased the overall mRNA m^6^A levels at 2 μM in NB4 cells. This effect is comparable to that of the FTO inhibitor FB23-2 at 5 μM. A treatment of FP54-NC, which retains the FTO-binding ability, also led to elevated m^6^A level in NB4 cells, although to a lesser extent when compared to that of FP54. In contrast, FP-1P did not induce any detectable increase in the overall mRNA m^6^A level, likely due to its limited cellular uptake (fig. S5).

### FTO degradation down-regulates pathways related to ribosome biogenesis in AML

**Fig. 3.**
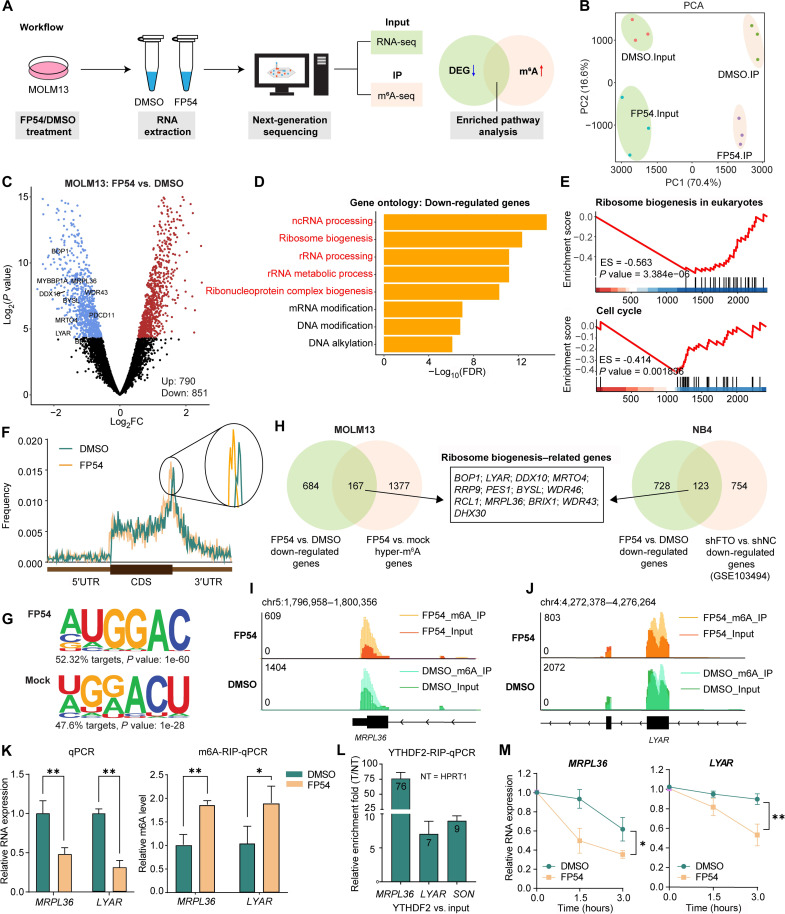
FTO degradation down-regulates ribosome biogenesis–related pathways in AML. (**A**) Schematic illustration of the RNA-seq and m^6^A-seq bioinformatic analyses strategy. (**B**) PCA of RNA-seq and m6A-RIP-seq data in MOLM-13 cells treated with 2 μM FP54 or DMSO for 48 hours (*n* = 3). (**C**) Volcano plot showing differentially expressed genes (DEG) after FP54 treatment. (**D**) GO analysis of down-regulated genes in MOLM-13 cells. ncRNA, noncoding RNA. (**E**) GSEA of ribosome biogenesis and cell cycle pathways. ES, enrichment score. (**F**) Metagene plot of m6A peak distribution in DMSO- and FP54-treated cells. 5′UTR, 5′ untranslated region. CDS, coding DNA sequence. (**G**) Motif analysis of m6A peaks. (**H**) Left: Overlap between down-regulated genes and m^6^A-hypermethylated genes in MOLM-13 cells. Right: Overlap of down-regulated genes in shFTO versus short hairpin negative control (shNC) NB4 cells (GSE103494). (**I** and **J**) Genome browser views of normalized read coverage for *MRPL36* and *LYAR* in input and IP samples. (**K**) Left: qPCR results for quantification of expression level of transcripts *MRPL36* and *LYAR* in MOLM-13 cells with FP54 or DMSO (vehicle) treatment. Right: m^6^A-RIP-qPCR results for quantification m^6^A level of transcripts *MRPL36* and *LYAR* in MOLM-13 cells with FP54 or DMSO (vehicle) treatment (*n* = 3). (**L**) Relative enrichment fold of *MPRL36*, *LYAR*, and *SON* in YTHDF2-RNA coimmunoprecipitation versus RNA-protein input control. Data were normalized to the enrichment of nontarget (NT) *HPRT1* and calculated as fold enrichment relative to input (*n* = 4). T, target. (**M**) Stability measurement of *MRPL36* and *LYAR* mRNA in MOLM-13 cells with FP54 or DMSO (vehicle) treatment (*n* = 3). *P < 0.05, **P < 0.01; unpaired Student’s *t* test. FDR, false discovery rate.

To better understand the underlying mechanisms on how FTO degrader inhibits AML cell proliferation, we performed RNA sequencing (RNA-seq) and m^6^A-RNA immunoprecipitation sequencing (m^6^A-RIP-seq) in dimethyl sulfoxide (DMSO)– or FP54-treated MOLM-13 (Fig. 3A). Principal components analysis (PCA) of both RNA-seq and m^6^A-RIP-seq data showed a clear separation between DMSO- and FP54-treated MOLM13 (Fig. 3B). RNA-seq data identified 1641 differentially expressed genes in FP54-treated cells consisting of 790 up-regulated and 851 down-regulated genes (Fig. 3C). Our RNA-seq data demonstrated that FTO degradation caused substantial suppression of MYC targets, E2F targets, and G_2_M checkpoint signal cascades as shown previously using FTO inhibitor FB23-2 (fig. S6, A and B) (*12*). In addition, our Gene Ontology (GO) analysis and gene set enrichment analysis (GSEA) revealed ribosome biogenesis and ribosomal RNA (rRNA) processing as among the top down-regulated pathways upon FTO deficiency (Fig. 3, D and E). GO enrichment analysis of up-regulated genes revealed significant up-regulation of biological processes related to chromatin remodeling, cellular stress responses, and immune pathways (fig. S6C), which is consistent with previous reports (*27*, *28*). Besides, GSEA also uncovered cell cycle–related genes significantly down-regulated after FTO degradation (Fig. 3E), which agrees with the growth inhibition effect caused by FP54 (Fig. 2A). Consistently, the FTO inhibitor FB23-2 also caused a down-regulated ribosome biogenesis pathway in NB4 cells based on our analysis (fig. S6, D and E).

m^6^A-RIP-seq data show that m^6^A peaks in mRNA of MOLM-13 cells were enriched at the start and stop codons (Fig. 3F) and were enriched in the canonical GGAC motif (Fig. 3G) (*29*). Compared to DMSO-treated cells, FP54-treated cells showed increased m^6^A enrichment in 3′ untranslated region (Fig. 3F). To further test whether the transcriptomic alterations related to ribosome biogenesis pathways are regulated by the FTO-mediated m^6^A demethylation, we intersected the significantly down-regulated and m^6^A-hypermethylated genes and obtained 167 transcripts (Fig. 3H). Consistent with the RNA-seq data, this group of 167 transcripts are the most enriched for the ribosome biogenesis process. Another set of RNA-seq data in FP54-treated NB4 cells shared a notable overlap in the down-regulated genes with a reported dataset in short hairpin RNA targeting FTO (shFTO) NB4 cells (Fig. 3H) (*12*). The shared down-regulated genes also show enrichment in ribosome biogenesis–related genes (*BOP1*, *LYAR*, *DDX10*, *MRTO4*, *RRP9*, *PES1*, *BYSL*, *WDR46*, *RCL1*, *MRPL36*, *BRIX1*, *WDR43*, and *DHX30*). The normalized read coverage for two representative genes *MRPL36* and *LYAR* for input and immunoprecipitation (IP) samples were shown in Fig. 3 (I and J). The expression levels and m^6^A levels of *MRPL36* and *LYAR* were validated by reverse transcription quantitative polymerase chain reaction (RT-qPCR) and m^6^A-RIP-qPCR, with results consistent with RNA-seq and m^6^A-RIP-seq data (Fig. 3K).

Since YTHDF2 is known to mediate mRNA decay through the m^6^A methylation (*29*), we performed YTHDF2-RIP-qPCR and identified *MRPL36* and *LYAR* mRNA as direct YTHDF2 targets. Enrichment of these transcripts was observed in YTHDF2 immunoprecipitates after normalized with the nontarget *HPRT1*, while a previously validated YTHDF2 target *SON* also showed robust enrichment (Fig. 3L) (*29*). Reduced stabilities of these two transcripts were observed when cells were treated with FP54 compared to those treated with DMSO (Fig. 3M). The degradation of FTO also led to the down-regulation of other ribosome biogenesis–related mRNAs (fig. S7A) and up-regulation of their m^6^A levels except for *BOP1* (fig. S7B). Some of these mRNAs were further confirmed to be targets of YTHDF2 (fig. S7C).

### FTO degradation by FP54 impairs translation process in AML

**Fig. 4.**
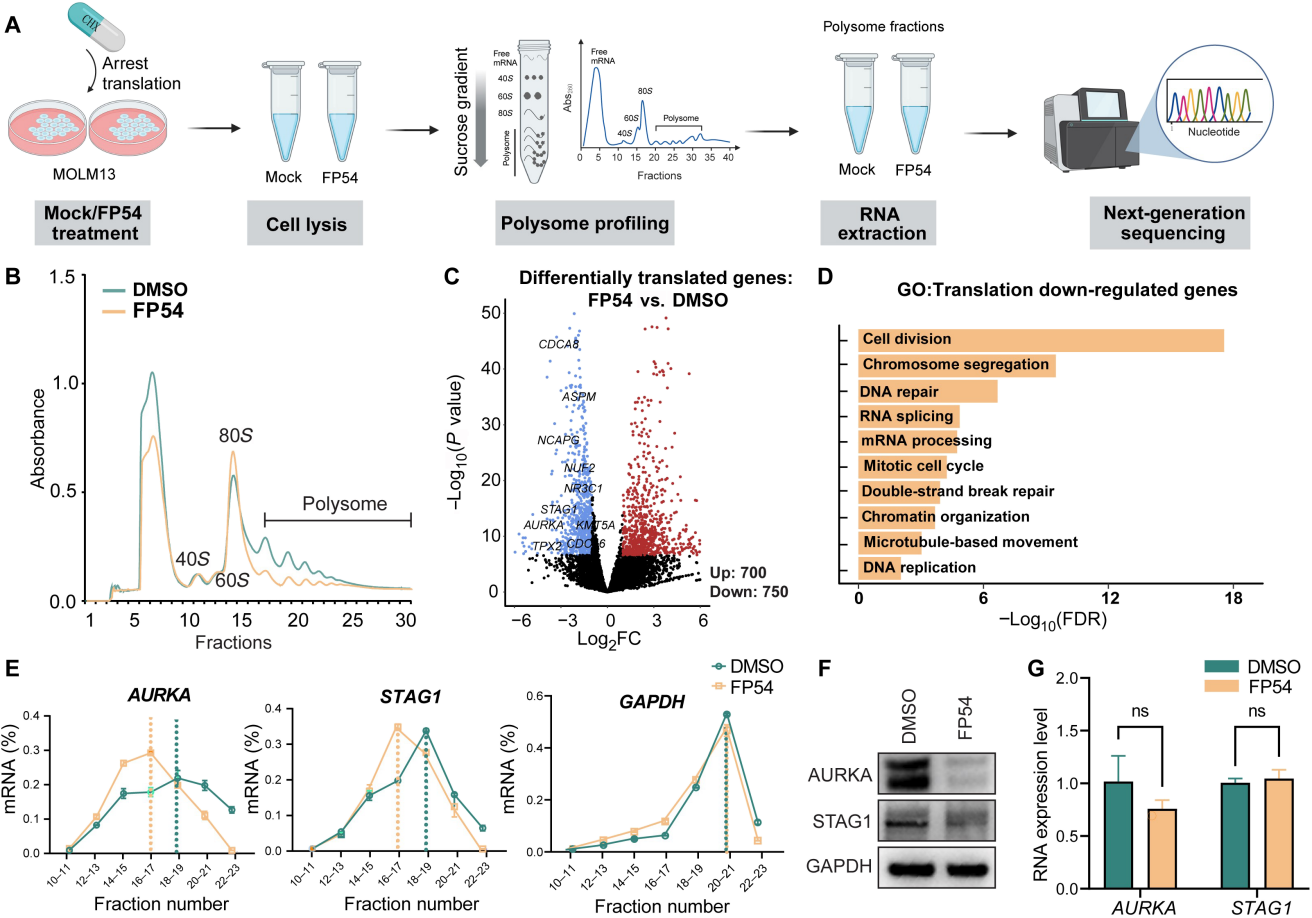
FTO degradation by FP54 impairs translation process in AML. (**A**) Workflow of polysome-sequencing for polysome fractions. (**B**) Polysome profiling in MOLM-13 cells treated with DMSO (vehicle) or FP54 (2 μM) for 48 hours. Absorbance was continuously measured at 254 nm. Data are representative of three independent samples. (**C**) Volcano blot of the alteration of gene expression in the polysome fractions of MOLM-13 cells treated with DMSO (vehicle) or FP54 (2 μM) for 48 hours. (**D**) GO analysis of TE down-regulated genes in the polysome fractions of MOLM-13 cells treated with DMSO (vehicle) or FP54 (2 μM) for 48 hours. (**E**) Quantification by RT-qPCR of *AURKA*, *STAG1*, and *GAPDH* mRNAs in every two polysome fractions, presented as a percentage of total mRNA, data were derived from *n* = 3 biologically independent samples. (**F**) Immunoblot of AURKA and STAG1 in MOLM-13 cells treated with DMSO (vehicle) or FP54 (2 μM) for 48 hours; blots are representative of three independent experiments. (**G**) RNA expressions of *AURKA* and *STAG1* in MOLM-13 cells treated with DMSO (vehicle) or FP54 (2 μM) for 48 hours; data were derived from *n* = 3 biologically independent samples, with unpaired Student’s *t* test.

Since the FP54-induced FTO degradation led to a substantial decrease in expression of mRNAs encoding ribosome biogenesis proteins, we performed polysome profiling to check the translation status with and without FP54 treatment (Fig. 4A). The FP54 treatment substantially reduced polysomes over 80*S* monosome, indicating a strongly inhibited translation process (Fig. 4B). To further investigate which transcripts were affected during translation, mRNA was extracted from the polysome fractions and analyzed using next-generation sequencing (Fig. 4B and fig. S8, A and B). Translation efficiency (TE) was calculated as the ratio of normalized read coverage in polysome mRNA over total mRNA (table S4). The RNA-seq data of the polysome fractions revealed 1450 genes with notably different TE between FP54- and DMSO-treated cells, comprising 700 TE up-regulated and 750 TE down-regulated genes (Fig. 4C).

GO analysis of the top 500 TE down-regulated genes revealed that FP54 treatment primarily affected genes involved in DNA replication and cell division (Fig. 4D), consistent with its inhibitory effect on cell growth. Among these genes, *AURKA* and *STAG1* play important roles in cell division by regulating chromosome segregation and mitotic spindle formation (*30*, *31*) and are considered promising targets for cancer therapy (*32*, *33*). We performed qPCR over different fractions to check the distribution of the *AURKA* and *STAG1* transcripts and found that the FP54 treatment notably shifted these two transcripts from the higher–molecular weight fractions to the lower–molecular weight fractions, while *GAPDH* mRNA remained unchanged, indicating inhibited translation of *AURKA* and *STAG1* (Fig. 4E). The protein expression levels of AURKA and STAG1 were also down-regulated as shown by Western blotting (Fig. 4F), while RNA expression levels of these two transcripts were not notably altered upon FP54 treatment (Fig. 4G), supporting a translational control mechanism by FTO degradation.

### Targeting FTO by FP54 inhibits AML development in mice

**Fig. 5.**
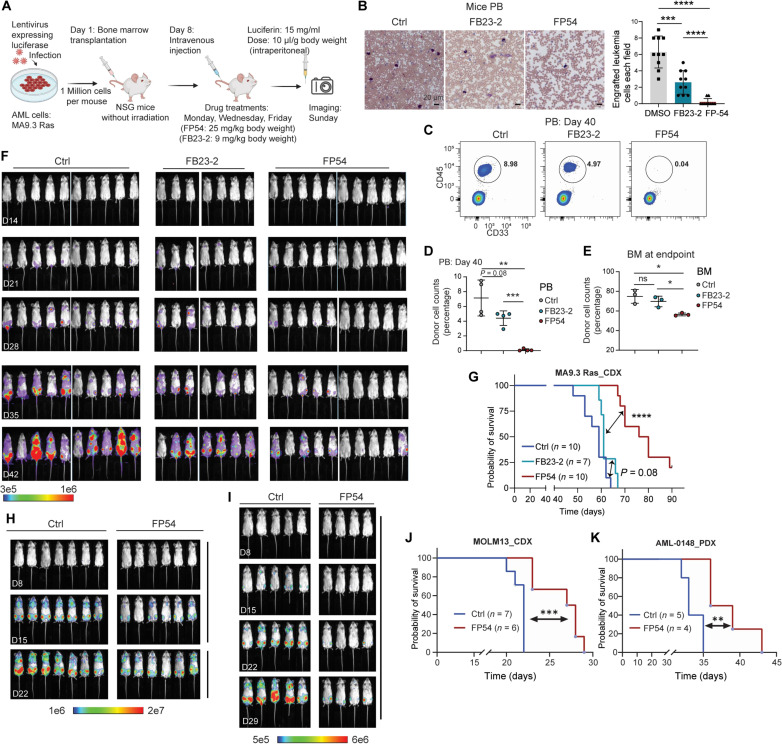
FP54 inhibits AML development in vivo. (**A**) Schematic diagram showing treatment strategy in mice. (**B**) Left: Wright-Giemsa staining for PB of AML cell transplanted mice (*n* = 10) after treatment with DMSO, FB23-2, or FP54 at the same time point. Scale bars, 20 μm. Right: Statistical analysis of Wright-Giemsa staining for PB of AML-transplanted mice (*n* = 10) after treatment with DMSO, FB23-2, or FP54 at the same time point, respectively. (**C**) Flow cytometry analysis for AML-transplanted mice PB. CD45 and CD33 were used as markers of human AML cell markers. (**D**) Statistical analysis of flow cytometry analysis for mice PB from different groups on day 40 after BM transplantation. Data were derived from at least three biologically independent samples. (**E**) Statistical analysis of flow cytometry analysis for mouse BM from different treatment groups at the endpoint of each sick mouse. Data were derived from at least three biologically independent samples. (**F**) In vivo bioluminescence imaging of xenotransplantation mouse models with AML cells (MA9.3Ras) expressing luciferase after treatments with DMSO, FB23-2, and FP54. D14, day 14. (**G**) Kaplan-Meier analysis of MA9.3Ras AML cells xenotransplanted NSG mice. (**H**) In vivo bioluminescence imaging of xenotransplantation mouse models with AML cells (MOLM13) expressing luciferase after treatment with DMSO or FP54. (**I**) In vivo bioluminescence imaging of xenotransplantation mouse models with AML cells (AML-0148) expressing luciferase after treatment with DMSO or FP54. (**J**) Kaplan-Meier analysis of MOLM13-xenotransplanted NSG mice. (**K**) Kaplan-Meier analysis of AML-0148–xenotransplanted NSG mice. For (B), (D), and (E), unpaired Student’s *t* test, *P < 0.05, **P < 0.01, ***P < 0.001, and ****P < 0.0001; for (G), (J), and (K), log-rank (Mantel-Cox) test, **P < 0.01, ***P < 0.001, and ****P < 0.0001.

To assess the therapeutic efficacy of FP54 in treating AML in vivo, we performed bone marrow (BM) transplantation in NSG (NOD-SCID IL2Rγ^null^) mice using the MA9.3Ras AML cells, which were generated by sequential retroviral expression of MLL-AF9 and NrasG12D cDNA into human umbilical cord blood CD34^+^ cells (*34*, *35*). MA9.3Ras AML cells transduced by lentivirus carrying luciferase were used as donor cells to be xeno-transplanted into immuno-compromised recipient mice, and then the leukemic mice were treated with DMSO, FP54, or FB23-2 (as a control) (Fig. 5A). Wright-Giemsa staining results showed that the leukemic cell engraftment in recipient mouse peripheral blood (PB) at the same time point was significantly decreased in the FP54-treated group compared to that in the FB23-2–treated group of mice or DMSO control group (Fig. 5B). We further performed flow cytometry analysis to detect the exact donor cell populations in mouse PB, and the data showed that FP54 treatment almost diminished the infiltration/engraftment of AML cells in PB (Fig. 5, C and D). Consistently, the BM engraftment of FP54-treated mice was significantly lower than that of DMSO- or FB23-2–treated groups at the endpoint of sick mice (Fig. 5E). Notably, FP54 treatment markedly reduced the leukemia burden and delayed the onset of AML cells in mouse models (Fig. 5F). The overall survival time of FP54-treated mice was largely prolonged (with 20% of recipient mice survived) compared to that in the DMSO or FB23-2 group (Fig. 5G), indicating much improved efficacy when targeting FTO with FP54 compared to previously reported inhibitor FB23-2. We tested two additional derivatives, **5** and **7**. They also led to reduced leukemia burden and delayed AML onset in vivo although they were less potent than FP54 (fig. S9).

Furthermore, in an independent xenograft model where NRGS (NOD-Rag1^−/−^IL2Rγ^null^ Sirpα^NOD^) mice were transplanted with MOLM13 cells expressing luciferase, the treatment of FP54 also notably reduced the leukemia burden, delayed disease onset, and prolonged survival in the treated mice (Fig. 5, H and J). Moreover, FP54 inhibited proliferation of three AML patient-derived xenograft (PDX) cell lines (fig. S10A). FP54 selectively degraded FTO (fig. S10, B to D) and induced apoptosis in AML-0148 cells and MA9.3RAS cells (fig. S10E). In the AML-0148 PDX model, FP54 treatment also notably delayed disease onset (Fig. 5I), reduced BM engraftment (fig. S11A), and extended overall survival (Fig. 5K), further supporting its translational potential as a therapeutic FTO degrader in AML.

FP54 did not affect body weight in any of the three AML mouse models during the treatment period (fig. S11, B to D), suggesting good tolerability. Complete blood count analysis of PB showed no significant changes in hematologic parameters or organ (heart, liver, spleen, lung, and kidney) weight following FP54 treatment (fig. S11E). In addition, histological examination revealed minimal effects on major organs (fig. S12). Collectively, these results indicate that FP54 has a favorable in vivo safety profile. Together, our data suggest that FP54 exhibits desirable therapeutic efficacy and a favorable safety profile in targeting AML initiation and progression in vivo.

## DISCUSSION

PROTAC technology has emerged as a successful strategy for selective degradation of disease-causing proteins (*36*). Unlike traditional inhibitors, PROTAC molecules act in a substoichiometric fashion, a key feature that notably enhances their therapeutic potential. In addition, the formation of PROTAC-induced ternary complex between the E3 ligase, the PROTAC, and the protein of interest (POI) allows for selective protein degradation. This selectivity can be finely tuned to target specific cell types or tissues, potentially reducing toxicity by specifically degrading the POI in tumor cells but not in healthy cells (*26*, *37*). In the present study, we report a potent VHL-based FTO degrader, FP54, designed through rational design and prodrug strategies. FP54 selectively degrades FTO in AML cells but not in normal cells such as hPBMCs, and it exhibits superior in vivo efficacy compared to its parent FTO inhibitor FB23-2. A recently reported FTO degrader, QP73, also induces FTO degradation and exhibits cancer cell killing activity in the nanomolar range (*13*). Both QP73 and our compound FP54 are based on the same FTO-binding scaffold derived from FB23-2, highlighting its utility of FTO binder for PROTAC design. Unlike QP73, which uses a CRBN ligand, FP54 is designed to recruit the VHL E3 ligase. These complementary strategies enrich the toolbox for probing FTO biology and further support the therapeutic potential of FTO-targeting PROTACs in AML.

Although FTO has been identified to be overexpressed and oncogenic in various cancers (*11*, *38*–*40*), it is expressed in most tissues and play roles in regulating metabolism and energy homeostasis (*41*). This broad expression suggests that tumor-selective FTO degradation induced by FP54 may have notable clinical benefits.

Dysregulated mRNA translation is a hallmark of cancer, typically driven by oncogenic pathways, making it an attractive target for anticancer therapies to overcome intratumor heterogeneity (*42*). As the key component of translation, ribosome biogenesis ensures the production of functional ribosomes required for protein synthesis, linking it directly to cellular growth and cancer progression (*17*). Targeting ribosome biogenesis represent a potential therapeutic strategy for cancer treatment (*21*, *43*). As the most prevalent RNA modifications in mRNA, m^6^A has been shown to influence various cellular processes. Studies on effects of m^6^A on ribosome biogenesis have been limited (*44*, *45*). We show here that FTO degradation increases m^6^A modifications on ribosome biogenesis–related mRNAs, promoting their YTHDF2-mediated degradation. Leukemia cells appear to hijack FTO demethylation to elevate ribosome biogenesis, the disruption of which impairs the whole cell translation, particularly the translation of DNA replication–related genes, ultimately inhibiting AML progression. Collectively, our findings provide a valuable tool compound for studying FTO’s regulatory functions and uncover a role of RNA m^6^A demethylation in ribosome biogenesis.

## MATERIALS AND METHODS

### Human cell lines

Human NB4, MOLM-13, SKM1, KOCL-69, NOMO-1, OCI-AML3, and ML-2 AML cell lines and peripheral blood mononuclear cells (PBMCs) used in this study were all purchased from ATCC (the American Type Culture Collection). NB4 cells were grown in RPMI 1640 media (Gibco) supplemented with 10% heat-inactivated fetal bovine serum (FBS) and 1% 100× Penicillin/Streptomycin (Pen/Strep; Gibco). MOLM-13, SKM1, KOCL-69, NOMO-1, OCI-AML3, ML-2, and PBMC cell lines were grown in RPMI 1640 media (Gibco) supplemented with 10% heat FBS and 1% 100× Pen/Strep (Gibco) at 37°C in 5% CO_2_. hPBMCs were activated with anti-CD3/CD28 beads and maintained at 0.5 to 2 × 10^6^ cells/ml before use. MA9.3, MA9.6, MA9.3Ras, MA9.6RAS, MA9.6ITD, AML-0148, HTB20-415, and 2016-35 AML cells (table S3) were cultured in Iscove’s modified Dulbecco’s medium supplemented with 20% FBS and stem cell factor (SCF; 10 ng/ml), thrombopoietin (TPO), Flt-3L, interleukin-3 (IL-3), and IL-6. Human embryonic kidney 293T cells are cultured in high-glucose Dulbecco’s modified Eagle’s medium (DMEM) containing sodium pyruvate and l-glutamine (Thermo Fisher Scientific) supplemented with 10% FBS and 1% 100× Pen/Strep (Gibco). MA9.3Ras AML cells were generated by sequential retroviral expression of MLL-AF9 and NrasG12D cDNA into human umbilical cord blood CD34^+^ cells as previously publications (*34*, *35*).

### Western blot

Cells were homogenized in CelLytic buffer (Sigma-Aldrich) containing 1× protease inhibitor cocktail (Roche) on ice for at least 15 min. The lysates were centrifuged, and the protein concentrations of supernatants were quantified using Pierce BCA Protein Assay Kits (Thermo Fisher Scientific). All samples were normalized and boiled at 95°C with 4× loading buffer (Bio-Rad) for 5 min. Samples were loaded into 4 to 12% NuPAGE bis-tris gel (Life Technologies) and transferred to nitrocellulose membranes (Bio-Rad). Membranes were blocked in 5% milk in tris-buffered saline with Tween-20 for 45 min at room temperature, incubated in a diluted primary antibody solution at 4°C overnight, washed, and incubated in a dilution of secondary antibody conjugated to horseradish peroxidase for 1 hour at room temperature. Protein bands were detected with SuperSignal West Femto Maximum Sensitivity Substrate (Thermo Fisher Scientific) on a FluroChem R (ProteinSimple). The intensity of each band was measured using ImageJ and quantified on the basis of the area of the intensity plot. Antibodies are listed in table S2.

### Cell proliferation assay

Cells were seeded in 96-well microplates at a density of 5000 cells per well. Compounds with serial 1:2 dilution in DMSO (1000×) was diluted for 50-fold with the cell culture medium. The prepared compound solution (5 μl) was added to the cell solution (95 μl) in triplicate. The plates were incubated for 3 days at 37°C and 5% CO_2_, and the cell viability was measured by the MTS Assay Kit (Cell Proliferation). The average cell viability values for each sample were calculated by setting the DMSO wells at 100%. The cures were fitted, and IC_50_ values were calculated using GraphPad Prism 9.

### Molecule docking

Ligands were built using Chemdraw software and optimized using LigPrep in Schrödinger 2018 software. The complexed x-ray structure of FTO (PDB: 6AKW) was downloaded from the Protein Data Bank and prepared with the Protein Preparation module. The docking grid was centered on the FB23 binding site, and the box size was set as 20 Å. The docking was performed according to the protocol present in Schrodinger Suite 2018 (*46*).

### Annexin V–FITC/propidium iodide apoptosis assay

For flow cytometry analysis of annexin V binding apoptosis assay, cells were seeded in six-well plates at a density of 50 × 10^4^ cells/ml, and DMSO or compound was added at the indicated concentrations. After 48 hours, the cells were collected and stained using the Annexin V Apoptosis Detection Kit FITC (fluorescein isothiocyanate) Kit (eBioscience) according to the manufacturers’ protocol. Then, 400 μl of binding buffer was added to samples, apoptosis was detected by flow cytometry, and results were analyzed with FlowJo V10 software.

### Differentiation assay

Cells were seeded in six-well plates at a density of 50 × 10^4^ cells/ml, and DMSO or compound was added at the indicated concentrations. After 72 hours, cells were collected and washed with phosphate-buffered saline (PBS) and incubated with 1% bovine serum albumin coupled with CD11b-phycoerythrin (BD Biosciences) and CD14-FITC (BD Biosciences) for 60 min on ice. After incubation, CD11b and CD14 expression levels were detected by flow cytometry, and the results were analyzed with FlowJo V10 software.

### Methylcellulose assay

Methylcellulose assays were performed in methylcellulose-based media (R&D Systems) according to the manufacturers’ protocol. Briefly, 1000 cells were seeded in the methylcellulose-based media in a six-well plate. For MA9.3Ras AML cells, the media was supplemented with SCF, TPO, Flt-3L, IL-3, and IL-6. The plates were incubated at 37°C and 5% CO_2_ for 14 days. Colony numbers were counted using a scoring grid using an inverted microscope.

### Cellular thermal shift assay

CETSA was performed to examine the cellular target engagement of FP-1P. Briefly, NB4 cells were incubated with various concentrations of FP-1P for 3 hours at 37°C. Then, the cells were collected by centrifugation, washed with PBS, and suspended in PBS containing the protease inhibitor cocktail. Subsequently, the cells were incubated for 3 min at 60°C and lysed by repeated freezing and thawing. Last, cell lysates were centrifuged at 4°C, and the supernatants were analyzed by Western blot using the FTO antibody and the glyceraldehyde phosphate dehydrogenase (GAPDH) antibody.

### RNA-seq and Me-RIP sequencing

NB4 or MOLM-13 cells were incubated with DMSO or FP-54 (2 μM) for 48 hours at 37°C and 5% CO_2_, and cells were collected by centrifugation, washed with PBS, and suspended in TRIzol reagent (Invitrogen). Total RNAs were extracted following the manufacturer’s protocol through ethanol precipitation. For mRNA enrichment, mRNA was purified with two rounds of polyadenylated RNA (polyA+) purification with a Dynabeads mRNA DIRECT kit (Ambion) following the manufacturer’s protocol. RNA concentration was measured using NanoDrop 1000 (Thermo Fisher Scientific). One microgram of mRNA for each sample was fragmented and enriched by the m^6^A antibody (Millipore). Fragmented mRNA (as “input”) and immunoprecipitated RNA (as “IP”) were subjected to RNA library construction following the manufacturer’s instructions of SMARTer Stranded Total RNA-Seq Kit v2–Pico Input Mammalian (Takara). All libraries were sequenced on Illumina NextSeq 500 with single-end 80-bp read length.

### Polysome profiling

Polysome profiling was performed according to the procedure we reported previously (*47*). Briefly, 20 million MOLM-13 cells were incubated with FP-54 (2 μM) for 48 hours. Cycloheximide (CHX) was added to the media at 100 mg/ml and incubated at 37°C for 7 min. Cells were collected by centrifugation and washed with ice-cold PBS with CHX (100 mg/ml), and the cell pellet was suspended in lysis buffer which was formulated as 20 mM Hepes (pH 7.6), 100 mM KCl, 5 mM MgCl_2_, CHX (100 μg/ml), 1% Triton X-100, freshly added 1:100 protease inhibitor (Roche), and SUPERasin (40 U/ml; Ambion). The lysate was loaded onto the sucrose gradient and centrifuged at 4°C at 28,000 rpm (Beckman, rotor SW28) for 3 hours. The samples were then fractioned, and the *A*_260_ absorbance of each fraction was analyzed. Total RNAs of polysome fractions were suspended in TRIzol LS Reagent (Invitrogen), RNA was extracted using the RNA Clean & Concentrator (Zymo), mRNAs were then purified with two rounds of polyA+ purification with a Dynabeads mRNA DIRECT kit (Ambion), and the libraries were constructed using the SMARTer Stranded Total RNA-Seq Kit v2–Pico Input Mammalian (Takara). All libraries were sequenced on Illumina NextSeq 500 with single-end 80-bp read length. For RT-qPCR analysis, every two fractions were combined, and total RNA was extracted. Relative RNA abundance was then quantified by RT-qPCR.

### Protein coimmunoprecipitation

MOLM13 cells were collected, resuspended with 2 volumes of lysis buffer [150 mM KCl, 10 mM Hepes (pH 7.6), 2 mM EDTA, 0.5% NP-40, 0.5 mM dithiothreitol (DTT), 1:100 protease inhibitor cocktail, and ribonuclease (RNase) inhibitor (400 U/ml)], and incubated on ice for 10 min. The lysate solution was centrifuged at 15,000*g* for 15 min at 4°C. YTHDF2 antibody was preimmobilized with protein A beads (Invitrogen) for 1 hour at 4°C. While 50 μl of cell lysate was saved as the input, the rest was incubated with the preimmobilized with protein A beads for 2 hours at 4°C. The beads were then washed with ice-cold NT2 buffer [200 mM NaCl, 50 mM Hepes (pH 7.6), 2 mM EDTA, 0.05% NP-40, 0.5 mM DTT, and RNase (200 U/ml) inhibitor] four times. Total RNA in the input and eluted (IP) samples were extracted and analyzed by RT-qPCR. Primer sequences are listed in table S1.

### Reverse transcription quantitative polymerase chain reaction

RT-qPCR was used to assess the relative abundance of RNA. Total RNA was reverse transcribed with PrimeScript RT Master Mix (Takara) to obtain cDNA. qPCR was performed by using FastStart Essential DNA Green Master (Roche) in machine LightCycler 96 (Roche). 18*S* rRNA was used as internal controls. When the external control is needed, 1 μl of 1:50 to 1:200 diluted non-m^6^A spike-in from the EpiMark *N*^6^-methyladenosine Enrichment Kit was added to each sample.

### RNA stability by qPCR

MOLM-13 cells were incubated with DMSO or FP-54 (2 μM) for 24 hours at 37°C and 5% CO_2_. Actinomycin D (2 μM) was added for 0, 1.5, and 3 hours. Cells were collected by centrifugation, washed with PBS, and suspended in TRIzol reagent (Invitrogen). Total RNAs were extracted using RNA Clean & Concentrator (Zymo) and then analyzed by RT-qPCR.

### RNA-seq, m^6^A-seq, and polysome sequencing data analysis

For RNA-seq analysis, raw reads were trimmed by Cutadapt (version 4.8) (*48*) to remove low-quality bases and adapters. Trimmed reads were subjected to Clumpify (BBMap package) (*49*) to remove duplicates. The deduplicated reads were aligned to the human genome (hg38 from UCSC) using STAR (Spliced Transcripts Alignment to a Reference; version 2.7.11b) (*50*) using default parameters. FeatureCounts software (version 2.0.3) (*51*) was used to count reads mapped to protein-coding genes from hg38 Gencode V42, and differentially expressed gene analysis was conducted by DEseq2 software. Differentially expressed genes were identified with a cutoff of *P* < 0.01 and |log_2_ fold change (FC)| ≥ 0.5.

For m^6^A-seq analysis, raw reads for both input and IP samples were trimmed by Cutadapt (version 4.8) (*48*) to remove low-quality bases and adapters. Trimmed reads were subjected to Clumpify (BBMap package) (*49*) to remove duplicates. The deduplicated reads were aligned to the human genome (hg38 from UCSC) using STAR (version 2.7.11b) (*50*) using default parameters. Mapped reads were separated by strands with SAMtools (version 1.21) (*52*), using “samtools view -f 83 (and 163)” for the forward strand and “samtools view -f 99 (and 147)” for the reverse strand. m^6^A peaks on each strand were called using MACS2 (*53*) with the parameter “--nomodel” separately. Significant peaks with *q* < 0.01 identified by MACS2 were considered.

For polysome-sequencing analysis, raw reads were trimmed by Cutadapt (version 4.8) (*48*) to remove low-quality bases and adapters. Trimmed reads were subjected to Clumpify (BBMap package) (*49*) to remove duplicates. The deduplicated reads were aligned to the human genome (hg38 from UCSC) using STAR (version 2.7.11b) (*50*) using default parameters. FeatureCounts software (version 2.0.3) (*51*) was used to count reads mapped to protein-coding genes from hg38 Gencode V42. TE was calculated as reads per kilobase of transcript per million mapped reads (RPKM; from polysome-sequencing) divided by RPKM (from RNA-seq). Differentially translated genes were identified with a cutoff of *P* < 0.05 and |log_2_(TE FC)| ≥ 1.

GO analysis was conducted using the Database for Annotation, Visualization, and Integrated Discovery. For regular RNA-seq, down-regulated genes (*P* < 0.01, |log_2_FC| ≤ −0.5) were subjected to GO enrichment analysis. For polysome-sequencing, differentially translated genes [*P* < 0.05, |log_2_(TE FC)| ≤ −1] were analyzed similarly.

GSEA was performed using the R package clusterProfiler (version 4.14.6) (*54*). A set of 2415 genes from regular RNA-seq, filtered by a cutoff of *P* < 0.1, was analyzed for enrichment in the gene sets “Ribosome biogenesis in eukaryotes” and “Cell cycle.”

### Animal model

All NOD.Cg-*Prkdc^scid^ Il2rg^tm1Wjl^*/SzJ (NSG, RRID: IMSR_JAX:005557) and NOD.Cg-*Rag1^tm1Mom^ Il2rg^tm1Wjl^* Tg (CMV-IL3, CSF2, KITLG) 1Eav/J (NRGS, RRID: IMSR_JAX:024099) mice breeding and drug treatment experiments were subject to institutional approval by the Beckman Research Institute Animal Care and Use Committee at City of Hope National Medical Center (#23066).

### Lentiviral constructs and transduction

Lentivirus was generated in 293T cells after cotransfecting the luciferase plasmid (pLenti-Luciferase-Neo) and lentiviral packaging helper mix (third generation contains pMD2.G, pMDLg/pRRE, and pRSV-Rev) using the transfection reagent (X-tremeGENE HP, MilliporeSigma) according to the manufacturer’s instructions. Twelve hours after the transfection, the medium was subsequently replaced by fresh DMEM medium with full growth nutrients. Twenty-four hours later, the supernatant was collected and filtered using a 0.45-μm filter. Fresh DMEM medium was immediately replaced, and the virus was collected again in another 24 hours. For the infection, 6 ml of the fresh virus supernatant was mixed into 3 million cells and then spin down for 2 hours (32°C, 1200 rpm). After infections, the cells were then transferred and cultured at 37°C in a humidified incubator with 5% CO_2_. AML cells transduced with the luciferase vector were selected using G418 sulfate (Thermo Fisher Scientific).

### Wright-Giemsa staining and histopathology analysis

Wright-Giemsa staining was performed using the Rapid-Chrome Kwik-Diff Staining System (Thermo Fisher Scientific, 9990702) according to the manufacturer’s instructions. Briefly, the slides of PB smears of recipient mice were dipped five to six times (1 s per dip) into methanol solution and then five to six times into eosin solution and methylene solution. The slides were then rinsed by dipping or swishing in distilled water followed by mounting with Epredia Mounting Medium (Thermo Fisher Scientific, 22-110-610) and covering with Epredia Gold Seal Cover Glasses (Thermo Fisher Scientific, 12-518-105B). The slides were subsequently imaged by Zeiss Observer 7 (Carl Zeiss Microscopy). For toxicity evaluation, portions of the heart, kidney, liver, muscle, spleen, and lung were collected from control and FP54-treated mice and fixed in formalin. Paraffin embedding, hematoxylin and eosin staining, and slide scanning were performed by the Pathology Core at City of Hope.

### Flow cytometry

About five drops (30 to 50 μl) of mice PB cells were collected after red cell lysis using ammonium chloride solution (STEMCELL Technologies, 07850) and filtration with 40-μm cell strainers. Then, the single cells were subjected to Fc receptor blocking (Thermo Fisher Scientific) within 20 μl of flow staining buffer (Thermo Fisher Scientific) for 10 min at room temperature followed by further staining with antibodies (CD45, Thermo Fisher Scientific, 17-9459-41; CD33, Thermo Fisher Scientific, 12-0339-42) for another 30 min kept from light in ice. The stained cells were washed with PBS one time and resuspended with PBS containing DAPI (0.75 μg/ml) to exclude dead cells. The flow cytometry assays were conducted with the LSR Fortessa flow cytometer (BD Biosciences). Flow cytometry data were analyzed with FlowJo (FlowJo LLC.).

### BM transplantation and bioluminescence imaging

One million AML cells (MA9.3Ras, MOLM13, and AML-0148) stably expressing firefly luciferase were intravenously injected into nonirradiated NSG (for MA9.3Ras) or sublethally irradiated (1.5 Gy) NRG-S (for MOLM13 and AML-0148) mice. For the MA9.3Ras model, equal molar amounts of compound FP54 (25 mg/kg body weight) or FB23-2 (9 mg/kg body weight) was intravenously injected into mice three times a week (Monday, Wednesday, and Friday). For MOLM13 and AML-0148 models, FP54 (25 mg/kg body weight) or PBS was intravenously injected into mice three times a week (Monday, Wednesday, and Friday). The progression of AML cells in recipient mice was monitored at the time points indicated using an in vivo imaging system of bioluminescence and fluorescence (Spectral Instruments Imaging, Tucson, AZ) every week. A total of 10 μl/g body weight of d-luciferin (GoldBio) in PBS (15 mg/ml) per mouse was injected 10 to 15 min before imaging. General anesthesia was induced with 5% isoflurane and continued during the procedure with 2% isoflurane introduced through a nose cone.

### Statistical analysis

Statistical analysis was performed using GraphPad Prism 9.1. Data are presented as means ± SD. No attrition or exclusion was applied unless otherwise noted.
