# Supplementary material for: FTO degrader impairs ribosome biogenesis and protein translation in acute myeloid leukemia
Source: Sci Adv. 2025 Aug 15;11(33):eadv7648. doi: 10.1126/sciadv.adv7648 (PMC12356235; doi:10.1126/sciadv.adv7648)
Supplement: Supplementary file 1 — Supplementary Methods Figs. S1 to S30 Tables S1 to S3 Legend for table S4 [file sciadv.adv7648_sm.pdf]

Science Advances
AAAS

Supplementary Materials for

FTO degrader impairs ribosome biogenesis and protein translation in acute myeloid leukemia

Wenlong Li *et al.*

Corresponding author: Chuan He, chuanhe@uchicago.edu; Jianjun Chen, jianchen@coh.org

Sci. Adv. **11**, eadv7648 (2025)
DOI: 10.1126/sciadv.adv7648

The PDF file includes:

Supplementary Methods
Figs. S1 to S30
Tables S1 to S3
Legend for table S4

Other Supplementary Material for this manuscript includes the following:

Table S4

Supplementary methods

Synthetic route for FTO degraders

Scheme S1

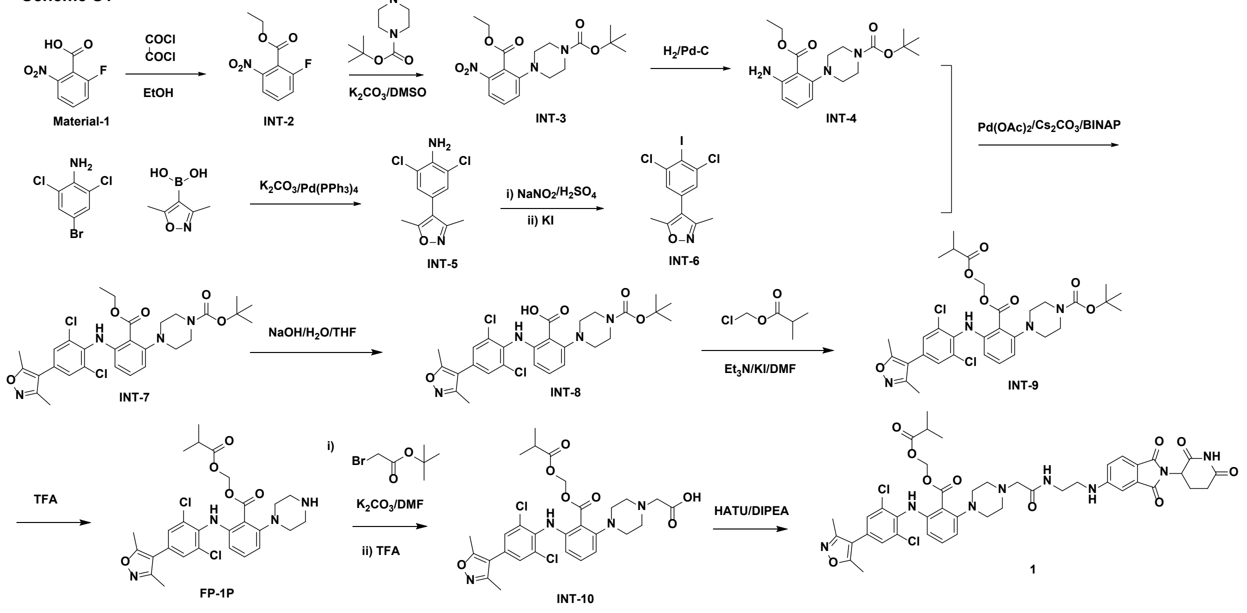

Synthesis of **INT-2**

To a solution of 2-fluoro-6-nitrobenzoic acid (10 g, 54 mmol) and a catalytic amount of DMF (395 mg) in anhydrous DCM (50 mL) was slowly added oxalyl chloride (5 mL, 59.4 mmol) at 0 °C, the mixture was stirred for 1 h at room temperature. The mixture was concentrated to give a residue, and was re-dissolved in DCM (50 mL), anhydrous EtOH (10 mL) was added and stirred for 1 h at room temperature. The mixture was concentrated to give a brown oil, which was purified by silica column chromatography (230-400 mesh), eluting with EA (5%-10%) in hexane, to afford the title compound as an oil. ^{1}H NMR (400 MHz, $CDCl_3$) δ 8.01 (dt, J = 8.3, 1.0 Hz, 1H), 7.61 (td, J = 8.3, 5.4 Hz, 1H), 7.50 (td, J = 8.3, 1.1 Hz, 1H), 4.50 (q, J = 7.2 Hz, 2H), 1.42 (t, J = 7.2 Hz, 3H); (ESI): m/z = 214.2 $[M + H]^+$.

Synthesis of **INT-3**

To a solution of ethyl 2-fluoro-6-nitrobenzoate (10 g, 47 mmol) and K_2CO_3 (7.8 g, 56 mmol) in DMSO (50 mL) was added tert-butyl piperazine-1-carboxylate (10.5 g, 56 mmol), the mixture was stirred 2 h at 80 ºC. The mixture was diluted with water, the organic layers was combined and washed by water (50 mL * 3) and brine (50 mL). The mixture was dried over anhydrous sodium sulfate, filtered, and concentrated. The obtained crude was purified by silica column chromatography (230-400 mesh), eluting with EtOAc (0-10%) in heptane, to afford the title

compound as a light yellow solid. ^{1}H NMR (400 MHz, $CDCl_3$) δ 7.97 (dd, J = 7.8, 1.4 Hz, 1H), 7.61 - 7.45 (m, 2H), 4.45 (q, J = 7.1 Hz, 2H), 3.62 - 3.50 (m, 4H), 3.07 - 2.78 (m, 4H), 1.49 (s, 9H), 1.41 (t, J = 7.1 Hz, 3H). (ESI): m/z =380.2 $[M + H]^+$.

Synthesis of **INT-4**

To a solution of (5 g, 13 mmol) in MeOH was added Pd-C (0.5 g), the solution was stirred at H_2 atmosphere overnight. After completion, the mixture was filtered, and the filtrate was concentrated to give a grey solid.

^{1}H NMR (400 MHz, $CDCl_3$) δ 7.12 (t, J = 8.1 Hz, 1H), 6.43 (ddd, J = 10.3, 8.1, 0.9 Hz, 2H), 4.39 (q, J = 7.1 Hz, 2H), 3.62 – 3.48 (m, 4H), 3.01 (s, 4H), 1.49 (d, J = 3.8 Hz, 9H), 1.41 (t, J = 7.1 Hz, 3H). (ESI): m/z =350.2 $[M + H]^+$.

Synthesis of **INT-5**

To the mixed solution of 4-bromo-2,6-dichloroaniline (10 g, 41 mmol) and (3,5-dimethylisoxazol-4-yl)boronic acid (7 g, 50 mmol) in dioxane (40 mL) and water (2 mL) was added K_2CO_3 (6.9 g, 50 mmol) and $Pd(PPh_3)_4$ (4.8 g, 0.4 mmol). The mixture was degassed for 10 min, and then stirred at 80 °C in N_2 atmosphere. After stirring for 4 h, the mixture the mixture filtered and the filtrate was concentrated to give a crude product, which was purified by silica column chromatography (230-400 mesh), eluting with EtOAc (5%-20%) in heptane, to afford the title compound as a white solid. ^{1}H NMR (400 MHz, $CDCl_3$) δ 7.07 (s, 2H), 4.55 (s, 2H), 2.38 (s, 3H), 2.24 (s, 3H). (ESI): m/z = 257.0 $[M + H]^+$.

Synthesis of **INT-6**

To the solution of 4-bromo-2,6-dichloroaniline (5 g, 19 mmol) in AcOH (20 mL), 10% H_2SO_4 aqueous (20 mL) was added in potions at 0 °C, $NaNO_2$ (1.6 g, 23 mmol) in water (2 mL) was added in potions, the mixture was stirred for 15 min. KI (3.9 g, 23 mmol) in water (2 mL) was added in potions. The mixture was stirred at room temperature for 2 h. The mixture was extracted with EA (100 mL), and washed by water (50) and brine (50 mL). The mixture was dried over anhydrous sodium sulfate, filtered, and concentrated. The obtained crude was purified by silica column chromatography (230-400 mesh), eluting with EtOAc (0-10%) to give a grey solid.^{1}H NMR (400 MHz, $CDCl_3$) δ 7.25 (s, 2H), 2.45 (s, 3H), 2.29 (s, 3H). (ESI): m/z = 371.0 $[M + H]^+$.

Synthesis of **INT-7**

The mixed solution of **INT-4** (2 g, 5.7 mmol), **INT-6** (2.5 g, 6.8 mmol), $Pd(OAc)_2$ (128 mg, 0.57 mmol), BINAP (356 mg, 0.57 mmol) and Cs_2CO_3 (2.2 g, 6.8 mmol) in dioxane (50 mL) was

degassed for 10 min, and then stirred at 100 °C in N_2 atmosphere. After stirring overnight, the mixture filtered and the filtrate was concentrated to give a crude product, which was then purified by silica column chromatography (230-400 mesh), eluting with DCM (2%-20%) in heptane, to afford the title compound as a white solid. ^{1}H NMR (400 MHz, $CDCl_3$) δ 7.30 (s, 1H), 7.25 (d, *J* = 1.0 Hz, 2H), 7.16 (t, *J* = 8.2 Hz, 1H), 6.61 (dd, *J* = 8.1, 1.0 Hz, 1H), 6.22 (d, *J* = 8.2 Hz, 1H), 4.50 – 4.36 (m, 2H), 3.54 (t, *J* = 5.0 Hz, 4H), 3.01 (t, *J* = 4.9 Hz, 4H), 2.44 (s, 3H), 2.30 (s, 3H), 1.49 (d, *J* = 1.0 Hz, 9H), 1.46 – 1.41 (m, 3H). (ESI): *m/z* = 589.2 $[M + H]^+$.

Synthesis of **INT-8**

To the solution of **INT-7** (1 g, 1.7 mmol) in MeOH (20 mL) was added 10% NaOH aqueous (20 mL), the mixture was stirred at 80 °C overnight. After completion, the solution was acidified by 10% HCl aqueous to pH 4, the precipitated solids were collected and dried to give 550 mg white solid. ^{1}H NMR (400 MHz, DMSO) δ 9.93 (s, 1H), 7.61 (s, 2H), 7.15 (t, *J* = 8.1 Hz, 1H), 6.79 (d, *J* = 7.9 Hz, 1H), 6.15 (d, *J* = 8.3 Hz, 1H), 3.59 – 3.45 (m, 4H), 3.07 (d, *J* = 10.1 Hz, 4H), 2.45 (s, 3H), 2.27 (s, 3H), 1.44 (s, 9H). (ESI): *m/z* = 561.2 $[M + H]^+$.

Synthesis of **INT-9**

To the solution of **INT-8** (0.5 g, 0.9 mmol), Et3N (180 mg, 1.78 mmol) in DMF (20 mL) was added chloromethyl isobutyrate (146 mg, 1.1 mmol), the mixture was stirred at 50 °C overnight. After completion, the mixture was diluted with water, the organic layers were combined and washed by water (50 mL * 3) and brine (50 mL). The mixture was dried over anhydrous sodium sulfate, filtered, and concentrated. The obtained crude was purified by silica column chromatography (230-400 mesh), eluting with EtOAc (10-30%) in heptane, to afford the title compound as a white solid. ^{1}H NMR (400 MHz, $CDCl_3$) δ 7.46 (s, 1H), 7.28 (d, *J* = 1.6 Hz, 2H), 7.20 (t, *J* = 8.2 Hz, 1H), 6.64 – 6.55 (m, 1H), 6.25 – 6.15 (m, 1H), 6.05 (d, *J* = 1.6 Hz, 2H), 3.59 (t, *J* = 5.0 Hz, 4H), 3.05 (t, *J* = 5.0 Hz, 4H), 2.62 (p, *J* = 7.0 Hz, 1H), 2.46 (d, *J* = 1.6 Hz, 3H), 2.32 (d, *J* = 1.5 Hz, 3H), 1.50 (d, *J* = 1.7 Hz, 9H), 1.19 (d, *J* = 7.0 Hz, 6H). (ESI): *m/z* = 661.2 $[M + H]^+$.

Synthesis of **FP-1P**

To the solution of **INT-9** (0.4 g, 0.6 mmol) in DCM (10 mL) was added TFA (2 mL), the solution was stirred at room temperature for 2 h. The mixture was concentrated to give a brown solid, which was used for next directly. ^{1}H NMR (400 MHz, Acetone) δ 7.56 (s, 2H), 7.25 (t, *J* = 8.2 Hz, 1H), 6.74 (dd, *J* = 8.1, 0.9 Hz, 1H), 6.19 (dd, *J* = 8.2, 0.9 Hz, 1H), 6.08 (s, 2H), 3.22 (d, *J* = 2.0 Hz, 8H),

2.72 – 2.60 (m, 1H), 2.49 (s, 3H), 2.30 (s, 3H), 1.16 (d, *J* = 7.0 Hz, 6H). ^{13}C NMR (101 MHz, Acetone) δ 175.29, 166.98, 166.09, 157.95, 152.92, 144.08, 134.96, 132.59, 132.18, 129.75, 129.39, 114.02, 113.61, 111.44, 109.92, 80.34, 51.58, 44.74, 33.51, 29.65, 29.59, 29.40, 29.20, 29.01, 28.82, 18.04, 10.73, 9.78. HR-MS $C_{27}H_{30}Cl_2N_4O_5$ calcd 560.1593, found (*m*/*z*) = 561.1632 $[M + H]^+$.

Synthesis of **INT-10**

To the solution of **FP-1P** (0.35 g, 0.6 mmol) and K_2CO_3 (0.10 g, 0.7 mmol) in DMF (10 mL) was added tert-butyl 2-bromoacetate (0.15 g, 0.7 mmol), the solution was stirred at 50 °C for 2 h. the mixture was diluted with water, the organic layers were combined and washed by water (20 mL * 3) and brine (20 mL). The mixture was dried over anhydrous sodium sulfate, filtered, and concentrated. The obtained crude was purified by silica column chromatography (230-400 mesh), eluting with EtOAc (10-30%) to give a 280 mg white solid, which was dissolve in TFA/DCM (1:5, 10 mL) and stirred at room temperature overnight. The mixture was concentrated to give a grey solid, which was used for next step directly.

Synthesis of compound **1**

To the solution of **INT-10** (20 mg, 0.03 mmol), 5-((2-aminoethyl)amino)-2-(2,6-dioxopiperidin-3-yl)isoindoline-1,3-dione hydrochloride (14 mg, 0.038 mmol), HATU (14 mg, 0.039 mmol) in DMF (5 mL) was added DIPEA (9.2 mg, 0.072 mmol), the mixture was stirred for 2 h. Then the mixture was extracted with ethyl acetate (20 mL * 3), and the organic layers was combined and washed by water (20 mL * 3) and brine (20 mL). The mixture was dried over anhydrous sodium sulfate, filtered, and concentrated. The obtained crude was purified by silica column chromatography (230-400 mesh), eluting with MeOH (2%-10%) in DCM to give a yellow solid, ^{1}H NMR (400 MHz, Acetone) δ 9.83 (s, 1H), 7.85 (d, *J* = 6.8 Hz, 1H), 7.58 (d, *J* = 8.3 Hz, 1H), 7.54 (s, 2H), 7.33 (s, 1H), 7.19 (t, *J* = 8.2 Hz, 1H), 7.06 (d, *J* = 2.2 Hz, 1H), 6.97 (dd, *J* = 8.3, 2.2 Hz, 1H), 6.60 (d, *J* = 8.1 Hz, 1H), 6.48 (t, *J* = 5.5 Hz, 1H), 6.11 (d, *J* = 8.2 Hz, 1H), 6.03 (s, 2H), 5.04 (dd, *J* = 12.6, 5.4 Hz, 1H), 3.60 – 3.43 (m, 4H), 3.05 – 3.02 (m, 4H), 2.96 – 2.88 (m, 1H), 2.79 – 2.69 (m, 3H), 2.68 – 2.58 (m, 5H), 2.47 (s, 3H), 2.29 (s, 3H), 2.14 (ddt, *J* = 12.6, 5.2, 2.4 Hz, 1H), 1.13 (d, *J* = 7.0 Hz, 6H). HR-MS $C_{44}H_{46}Cl_2N_8O_{10}$ calcd 916.2714, found (*m*/*z*) = 3917.2812 $[M + H]^+$.

Synthesis of compound **2**

Yellow solid, ^{1}H NMR (400 MHz, Acetone) δ 9.84 (s, 1H), 7.68 (s, 1H), 7.58 (d, *J* = 8.3 Hz, 1H), 7.56 (s, 2H), 7.35 (s, 1H), 7.18 (t, *J* = 8.1 Hz, 1H), 7.11 (d, *J* = 2.2 Hz, 1H), 6.59 (d, *J* = 8.1 Hz, 1H), 6.49 (s, 1H), 6.12 (d, *J* = 8.2 Hz, 1H), 6.05 (s, 2H), 5.05 (dd, *J* = 12.6, 5.4 Hz, 1H), 4.03 (s, 2H), 3.78 (t, *J* = 5.2 Hz, 2H), 3.61 (t, *J* = 5.3 Hz, 2H), 3.57 – 3.41 (m, 5H), 3.08 (dd, *J* = 10.4, 5.5 Hz, 6H), 2.79 – 2.58 (m, 7H), 2.49 (s, 3H), 2.31 (s, 3H), 2.22 – 2.10 (m, 1H), 1.15 (d, *J* = 7.0 Hz, 7H). HR-MS $C_{46}H_{50}Cl_2N_8O_{11}$ calcd 960.2976, found (*m*/*z*) = 961.2863 $[M + H]^+$.

Synthesis of compound **3**

Yellow solid, ^{1}H NMR (400 MHz, Acetone) δ 9.87 (s, 1H), 7.66 (d, *J* = 8.5 Hz, 1H), 7.56 (s, 2H), 7.40 – 7.30 (m, 2H), 7.27 (dd, *J* = 8.5, 2.4 Hz, 1H), 7.22 (t, *J* = 8.1 Hz, 1H), 6.68 (d, *J* = 8.1 Hz, 1H), 6.12 (d, *J* = 8.2 Hz, 1H), 6.07 (s, 2H), 5.08 (dd, *J* = 12.6, 5.4 Hz, 1H), 4.12 (d, *J* = 13.0 Hz, 2H), 3.13 – 3.02 (m, 6H), 2.88 – 2.71 (m, 4H), 2.70 – 2.52 (m, 5H), 2.49 (s, 3H), 2.36 – 2.28 (m, 5H), 2.19 (ddt, *J* = 13.2, 10.2, 4.3 Hz, 2H), 1.17 (dd, *J* = 7.0, 0.7 Hz, 6H); ^{13}C NMR (101 MHz, Acetone) δ 175.11, 171.77, 169.35, 167.73, 167.36, 167.04, 166.06, 157.94, 155.59, 153.19, 144.13, 135.14, 134.61, 132.58, 132.07, 129.66, 129.37, 124.76, 118.49, 117.77, 114.03, 112.80, 110.85, 108.93, 107.83, 80.15, 64.18, 53.70, 52.49, 49.16, 47.73, 33.55, 33.13, 31.15, 30.02, 29.70, 22.57, 18.09, 10.74. HR-MS $C_{46}H_{49}Cl_2N_7O_9$ calcd 960.2976, found (*m*/*z*) = 961.2863 $[M + H]^+$.

Synthesis of compound **5**

White solid, ^{1}H NMR (400 MHz, Acetone) δ 8.86 (s, 1H), 7.57 (d, *J* = 7.2 Hz, 4H), 7.54 – 7.45 (m, 3H), 7.39 – 7.28 (m, 2H), 7.25 (s, 1H), 6.87 (d, *J* = 8.9 Hz, 1H), 6.76 (d, *J* = 8.1 Hz, 1H), 6.22 (d, *J* = 8.2 Hz, 1H), 6.09 (s, 2H), 5.08 (t, *J* = 7.1 Hz, 1H), 4.69 (d, *J* = 9.0 Hz, 1H), 4.61 (t, *J* = 8.2 Hz, 1H), 4.49 (s, 1H), 3.94 (d, *J* = 11.1 Hz, 1H), 3.76 (dd, *J* = 11.0, 4.0 Hz, 2H), 3.32 (q, *J* = 6.5 Hz, 2H), 3.20 (t, *J* = 4.7 Hz, 4H), 3.12 (s, 2H), 2.80 – 2.65 (m, 5H), 2.60 (s, 3H), 2.54 (s, 3H), 2.38 (s, 4H), 2.23 – 2.11 (m, 2H), 1.70 (d, *J* = 7.6 Hz, 2H), 1.62 (d, *J* = 7.3 Hz, 2H), 1.55 (d, *J* = 7.0 Hz, 3H), 1.25 (d, *J* = 7.0 Hz, 6H), 1.11 (s, 9H). HR-MS $C_{57}H_{71}Cl_2N_9O_{10}S$ calcd 1143.4422, found (*m*/*z*) = 1144.4492 $[M + H]^+$.

Synthesis of FP-54 (**6**)

White solid, ^{1}H NMR (400 MHz, Acetone) δ 8.86 (s, 1H), 7.79 (d, *J* = 7.8 Hz, 1H), 7.56 (s, 3H), 7.52 – 7.42 (m, 4H), 7.37 (s, 1H), 7.22 (t, *J* = 8.2 Hz, 1H), 7.09 (d, *J* = 9.0 Hz, 1H), 6.68 (d, *J* = 8.1 Hz, 1H), 6.13 (d, *J* = 8.2 Hz, 1H), 6.06 (s, 2H), 5.08 (p, *J* = 7.1 Hz, 1H), 4.72 – 4.57 (m, 2H), 4.49 (s, 1H), 4.34 (s, 1H), 3.97 – 3.82 (m, 1H), 3.72 (dd, *J* = 10.8, 4.1 Hz, 1H), 3.24 (t, *J* = 6.7 Hz, 2H), 3.13 (t, *J* = 4.6 Hz, 4H), 3.06 (s, 2H), 2.74 – 2.58 (m, 5H), 2.49 (d, *J* = 4.9 Hz, 6H), 2.35 –

2.21 (m, 6H), 2.17 – 2.10 (m, 2H), 1.61 (d, *J* = 11.0 Hz, 2H), 1.53 (s, 2H), 1.48 (d, *J* = 7.0 Hz, 3H), 1.16 (d, *J* = 7.0 Hz, 6H), 1.04 (s, 9H). ^{13}C NMR (101 MHz, Acetone) δ 175.19, 172.67, 170.84, 170.57, 167.24, 166.09, 157.96, 153.01, 150.47, 148.27, 144.60, 144.16, 135.11, 132.61, 132.11, 131.36, 130.45, 129.68, 129.37, 129.11, 126.58, 126.54, 114.03, 112.97, 111.00, 109.19, 80.24, 69.58, 61.52, 58.95, 57.03, 56.50, 54.79, 53.51, 52.33, 48.33, 42.77, 38.44, 38.31, 37.15, 35.41, 35.14, 33.54, 29.60, 29.41, 29.22, 29.02, 28.49, 26.40, 26.08, 25.85, 25.58, 21.91, 18.09, 15.45, 12.23, 10.74. HR-MS $C_{59}H_{75}Cl_2N_9O_{10}S$ calcd 1171.4735, found (*m*/*z*) = 1172.4796 $[M + H]^+$.

Synthesis of **FP-54-NC**

White solid, ^{1}H NMR (400 MHz, Acetone) δ 8.87 (d, *J* = 2.5 Hz, 1H), 7.82 (d, *J* = 7.8 Hz, 1H), 7.55 (s, 3H), 7.51 – 7.44 (m, 5H), 7.36 (s, 1H), 7.20 (dt, *J* = 16.7, 8.7 Hz, 2H), 6.68 (d, *J* = 8.1 Hz, 1H), 6.13 (d, *J* = 8.2 Hz, 1H), 6.06 (s, 2H), 5.13 - 5.03 (m, 1H), 4.67 (dd, *J* = 11.8, 8.6 Hz, 2H), 4.51 – 4.45 (m, 1H), 4.02 – 3.87 (m, 2H), 3.72 (dd, *J* = 10.8, 4.1 Hz, 1H), 3.44 (t, *J* = 7.4 Hz, 1H), 3.25 (q, *J* = 6.7 Hz, 2H), 3.12 (t, *J* = 4.8 Hz, 4H), 3.06 (s, 2H), 2.77 - 2.56 (m, 5H), 2.49 (d, *J* = 4.8 Hz, 7H), 2.38 – 2.20 (m, 6H), 1.61 (d, *J* = 11.8 Hz, 3H), 1.54 (d, *J* = 6.5 Hz, 3H), 1.49 (dd, *J* = 6.9, 4.3 Hz, 13H), 1.15 (d, *J* = 7.0 Hz, 6H), 1.04 (s, 9H). HR-MS $C_{59}H_{75}Cl_2N_9O_{10}S$ calcd 1171.4735, found (*m*/*z*) = 1172.4762 $[M + H]^+$.

Synthesis of **7**

White solid, ^{1}H NMR (400 MHz, Acetone) δ 8.86 (d, *J* = 3.0 Hz, 1H), 7.73 (d, *J* = 7.8 Hz, 1H), 7.56 (s, 2H), 7.48 (s, 4H), 7.36 (s, 1H), 7.23 (q, *J* = 8.1 Hz, 2H), 7.01 (d, *J* = 9.1 Hz, 1H), 6.67 (d, *J* = 8.1 Hz, 1H), 6.13 (d, *J* = 8.2 Hz, 1H), 6.06 (s, 2H), 5.15 – 4.99 (m, 1H), 4.72 – 4.57 (m, 2H), 4.51 (s, 1H), 4.38 – 4.26 (m, 1H), 3.87 (d, *J* = 10.9 Hz, 1H), 3.74 (dd, *J* = 10.7, 4.4 Hz, 2H), 3.12 (t, *J* = 4.7 Hz, 4H), 3.02 (s, 2H), 2.74 – 2.58 (m, 5H), 2.49 (d, *J* = 4.5 Hz, 7H), 2.12 (s, 2H), 1.98 (s, 4H), 1.82 (d, *J* = 13.3 Hz, 1H), 1.66 – 1.54 (m, 3H), 1.49 (t, *J* = 6.4 Hz, 5H), 1.40 – 1.24 (m, 5H), 1.16 (d, *J* = 7.0 Hz, 6H), 1.04 (s, 9H). HR-MS $C_{59}H_{73}Cl_2N_9O_{10}S$ calcd 1169.4578, found (*m*/*z*) = 1168.3923 $[M - H]^-$.

Synthesis of compound **8**

White solid, ^{1}H NMR (400 MHz, Acetone) δ 8.69 (s, 1H), 7.65 (t, *J* = 6.0 Hz, 1H), 7.53 (d, *J* = 6.2 Hz, 1H), 7.40 (d, *J* = 6.8 Hz, 3H), 7.20 (s, 1H), 7.08 (dd, *J* = 9.1, 3.2 Hz, 1H), 6.99 – 6.91 (m, 2H), 6.88 (dd, *J* = 7.8, 1.7 Hz, 1H), 6.37 (dd, *J* = 8.1, 0.9 Hz, 1H), 5.95 (d, *J* = 8.1 Hz, 1H), 5.89 (s, 2H), 4.62 – 4.47 (m, 2H), 4.42 (dt, *J* = 11.5, 6.5 Hz, 2H), 4.28 (dd, *J* = 15.8, 5.6 Hz, 1H), 4.21 – 4.09 (m, 2H), 3.85 – 3.76 (m, 2H), 3.71 – 3.61 (m, 2H), 3.57 (t, *J* = 5.4 Hz, 2H), 3.34 (q, *J* = 5.1 Hz,

2H), 2.87 (dq, J = 9.1, 4.9 Hz, 6H), 2.55 – 2.44 (m, 5H), 2.34 (d, J = 3.2 Hz, 7H), 2.16 (s, 4H), 2.06 (dt, J = 8.3, 2.7 Hz, 3H), 1.23 – 1.07 (m, 6H), 1.00 (d, J = 7.0 Hz, 7H), 0.88 (s, 9H). HR-MS $C_{59}H_{72}Cl_2FN_9O_{12}S$ calcd 1219.4382, found (m/z) = 1220.4436 $[M + H]^+$.

Synthesis of compound **4**

To the solution of **INT-10** (20 mg, 0.035 mmol), VHL-C7-COOH (26 mg, 0.043 mmol), HATU (14 mg, 0.039 mmol) in DMF (5 mL) was added DIPEA (9.2 mg, 0.072 mmol), the mixture was stirred for 2 h. Then the mixture was extracted with ethyl acetate (20 mL * 3), and the organic layers was combined and washed by water (20 mL * 3) and brine (20 mL). The mixture was dried over anhydrous sodium sulfate, filtered, and concentrated. The obtained crude was purified by silica column chromatography (230-400 mesh), eluting with MeOH (2%-10%) in DCM to give a white solid, ^{1}H NMR (400 MHz, Acetone) δ 8.86 (s, 1H), 7.77 (d, J = 7.8 Hz, 1H), 7.56 (s, 2H), 7.53 – 7.43 (m, 4H), 7.37 (s, 1H), 7.23 (t, J = 8.2 Hz, 1H), 7.06 (d, J = 9.0 Hz, 1H), 6.69 (d, J = 8.0 Hz, 1H), 6.18 (d, J = 8.2 Hz, 1H), 6.08 (s, 2H), 5.08 (s, 1H), 4.73 – 4.55 (m, 2H), 4.49 (s, 1H), 4.30 (d, J = 3.8 Hz, 1H), 3.89 (s, 2H), 3.71 (dt, J = 16.8, 4.6 Hz, 5H), 3.47 (q, J = 7.5 Hz, 1H), 3.08 (d, J = 5.9 Hz, 2H), 3.00 (s, 2H), 2.96 (s, 1H), 2.68 – 2.56 (m, 1H), 2.49 (d, J = 3.4 Hz, 6H), 2.41 (dd, J = 8.4, 6.7 Hz, 2H), 1.68 - 1.56 (m, 4H), 1.50 (t, J = 7.9 Hz, 9H), 1.15 (d, J = 6.9 Hz, 6H), 1.05 (s, 9H). HR-MS $C_{59}H_{74}Cl_2N_8O_{10}S$ calcd 1156.4626, found (m/z) = 1157.4813 $[M + H]^+$.

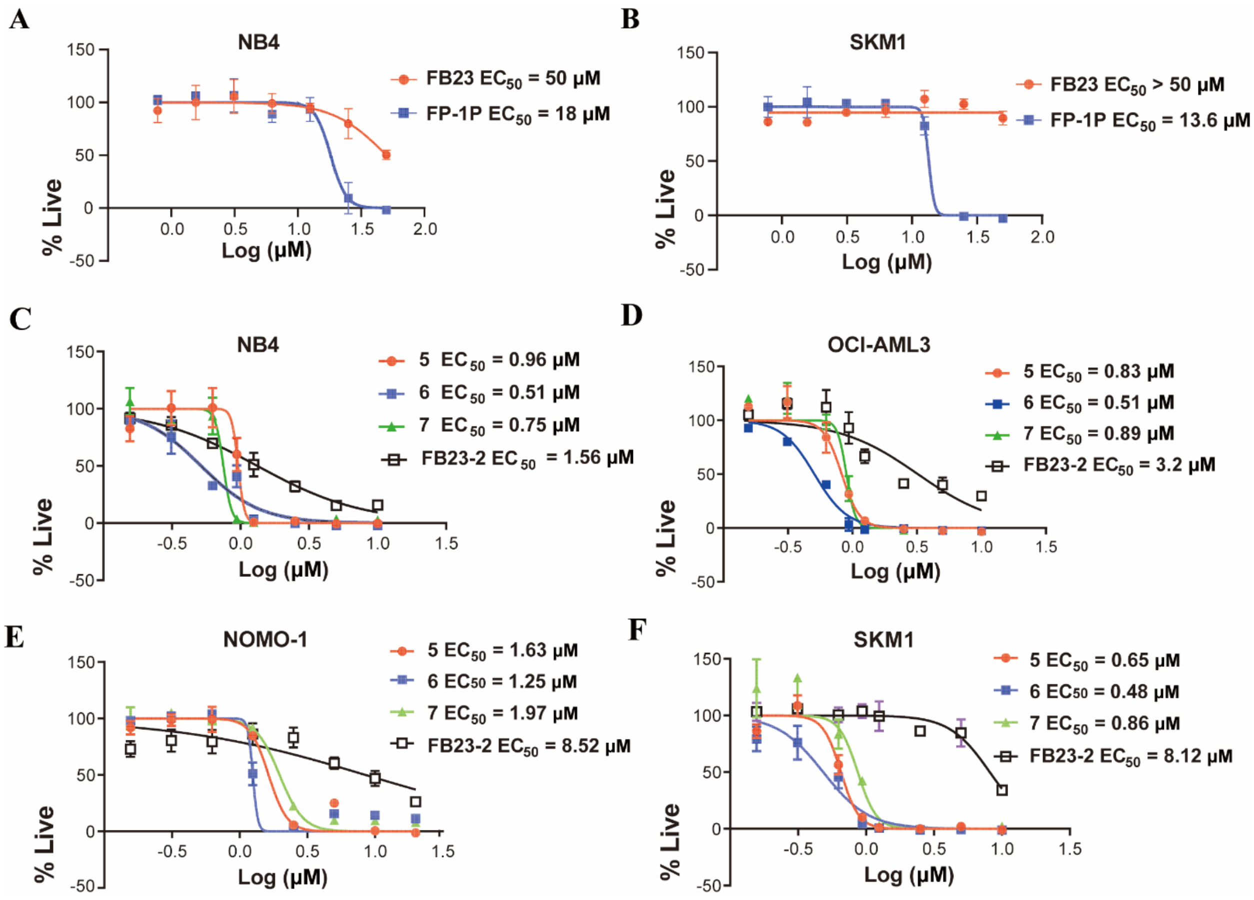

Figure S1. The anti-proliferative activities of designed compounds on AML cells.

(A) The anti-proliferative activity of compound FP-1P and FB23 in NB4 cells.

(B) The anti-proliferative activity of compound FP-1P and FB23 in SKM1 cells.

(C) The anti-proliferative activities of compounds **5-7** and FB23-2 in NB4 cells.

(D) The anti-proliferative activities of compounds **5-7** and FB23-2 in OCI-AML3 cells.

(E) The anti-proliferative activities of compounds **5-7** and FB23-2 in NOMO-1 cells.

(F) The anti-proliferative activities of compounds **5-7** and FB23-2 in SKM1 cells.

Values in the plots are shown as mean ± SD from n = 3 independent experiments.

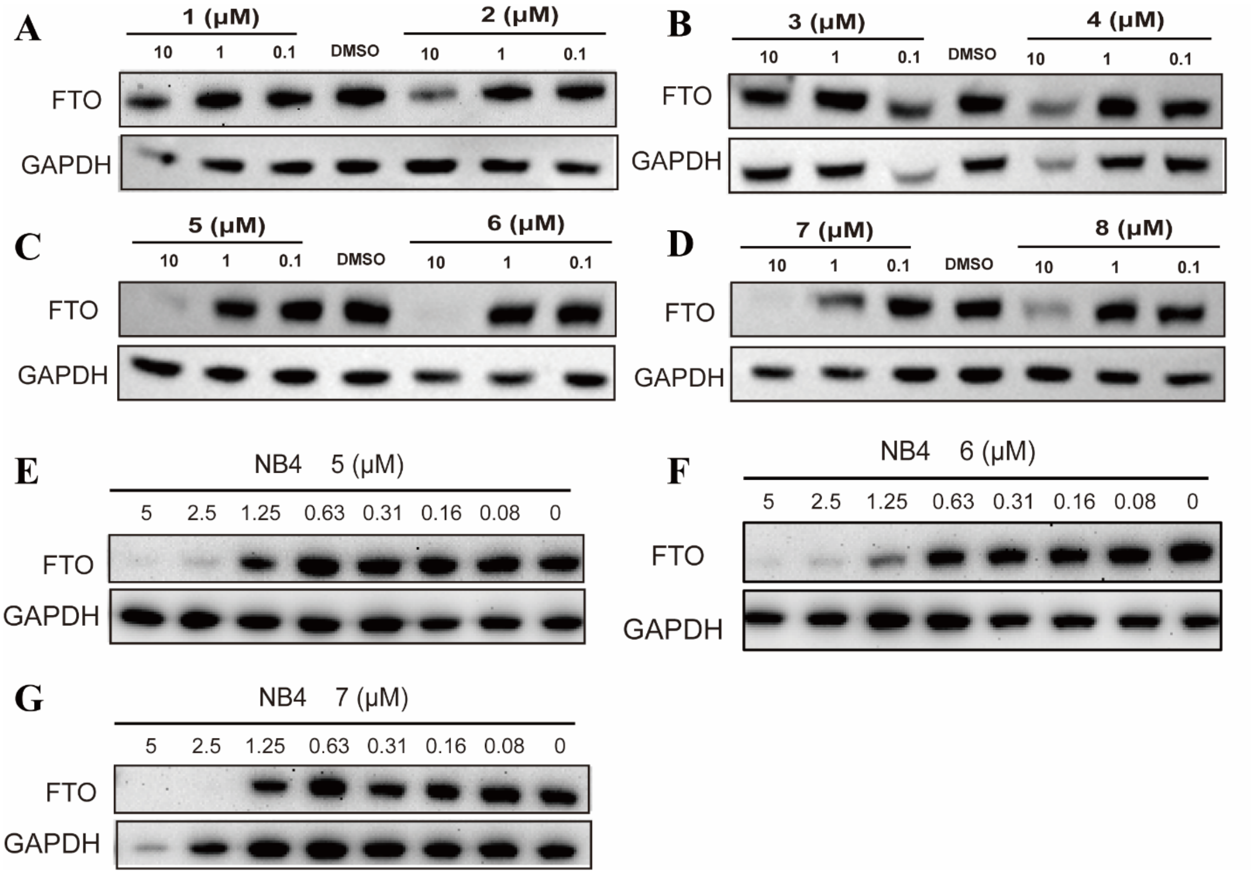

Figure S2. The FTO degradation activities of designed compounds.

(A) Immunoblots for FTO in NB4 cells treated with 0.1 μM, 1 μM and 10 μM of compounds **1**, **2** and DMSO for 48 h.

(B) Immunoblots for FTO in NB4 cells treated with 0.1 μM, 1 μM and 10 μM of compounds **3**, **4** and DMSO for 48 h.

(C) Immunoblots for FTO in NB4 cells treated with 0.1 μM, 1 μM and 10 μM of compounds **5**, **6** and DMSO for 48 h.

(D) Immunoblots for FTO in NB4 cells treated with 0.1 μM, 1 μM and 10 μM of compounds **7**, **8** and DMSO for 48 h.

(E) Immunoblots for FTO in NB4 cells treated with compound **5** at indicated concentrations for 48 h.

(F) Immunoblots for FTO in NB4 cells treated with compound **6** at indicated concentrations for 48 h.

(G) Immunoblots for FTO in NB4 cells treated with compound **7** at indicated concentrations for 48 h.

The blots shown are representative of three independent experiments.

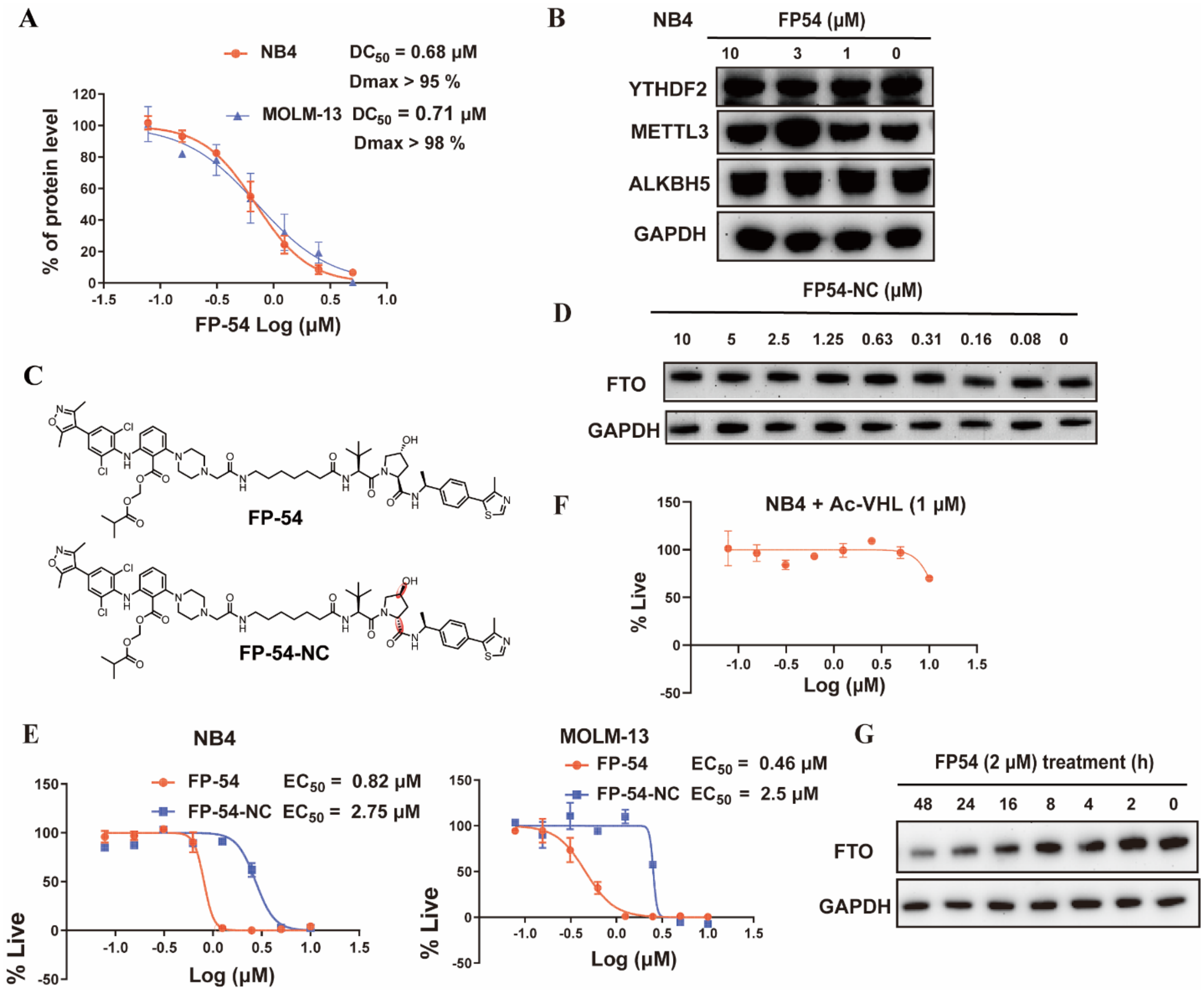

Figure S3. FP54 induces degradation of FTO through the ubiquitin-proteasomal system.

(A) DC_{50} and Dmax values of FP54 in NB4 and MOLM-13 cells after treatment for 48 h.

(B) Immunoblots for YTHDF2, METTL3, ALKBH5 and GAPDH in NB4 cells after treatment with FP54 at indicated concentrations for 48 h.

(C) Chemical structures of FP54 and negative control compound FP54-NC.

(D) Immunoblots for FTO and GAPDH in NB4 cells after treatment with FP54-NC at indicated concentrations for 48 h.

(E) Antiproliferative activities of FP54 and FP54-NC in NB4 and MOLM-13 cells.

(F) Antiproliferative activities of FP54 in NB4 cells in the presence of Ac-VHL (1 μM).

(G) Immunoblots for FTO and GAPDH in NB4 cells after treatment with FP54 (2 μM) for indicated time.

The blots shown in B, D and G are representative of 3 independent experiments. Values in the plots of E and F are shown as mean ± SD from $n = 3$ independent experiments.

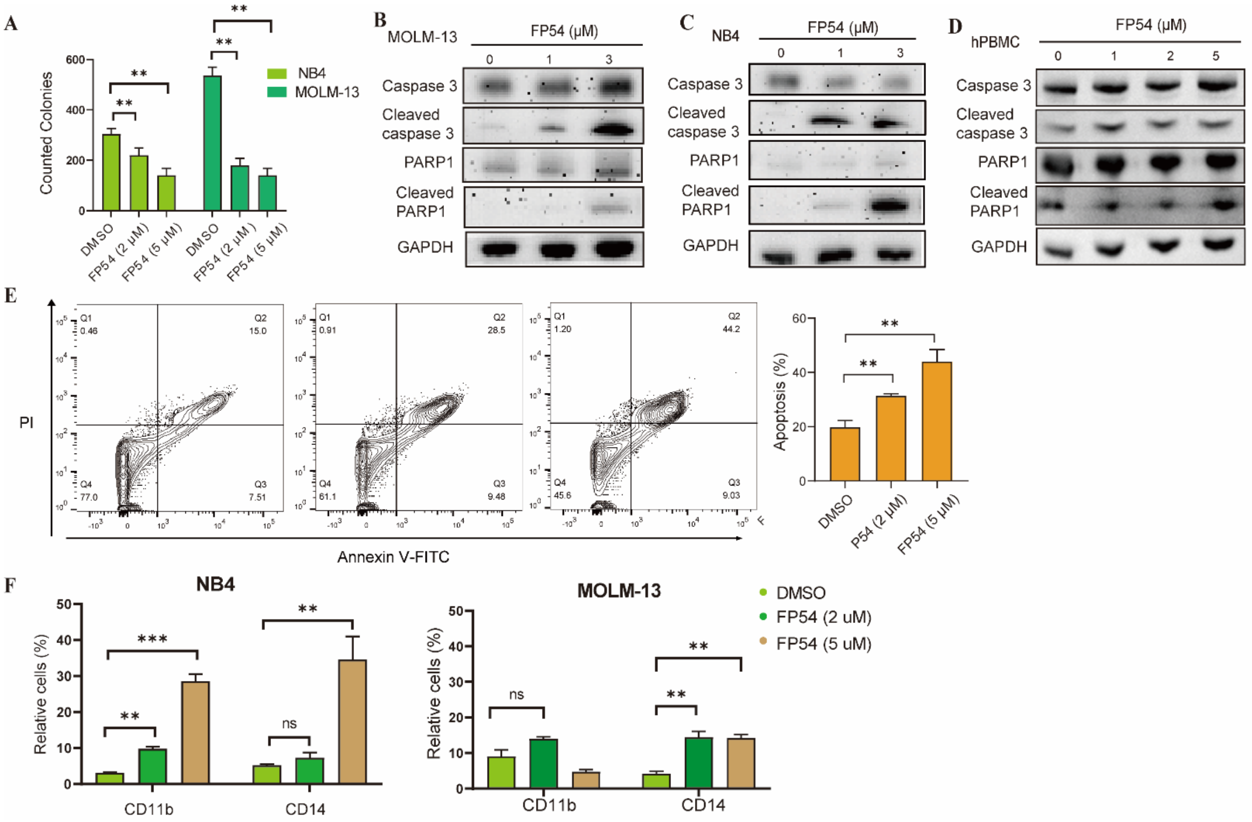

Figure S4. The effects of FP54 on NB4, MOLM-13 and hPBMC cells

(A) Methylcellulose colony forming assay after treatment of FP54 at 2 and 5 µM for 15 days.

(B) Immunoblots of apoptotic markers using immunoblotting after 48 h treatment of MOLM-13 cells with FP54 at 1 and 3 µM.

(C) Immunoblots of apoptotic markers using immunoblotting after 48 h treatment of NB4 cells with FP54 at 1 and 3 µM.

(D) Immunoblots of apoptotic markers using immunoblotting after 48 h treatment of hPBMC cells with FP54 at 1, 2 and 5 µM. Data shown in B, C, D are representative of three independent experiments.

(E) Apoptosis quantification in NB4 cells treated with DMSO and FP54 at indicated concentrations for 48 h (n = 3). The PI- and Annexin V-positive cells were quantified by flow cytometry.

(F) Analysis of the myeloid differentiation markers CD11b and CD14 in NB4 (Left) and MOLM-13 cells (Right) after 72 h treatment with FP54 at 2 and 5 μM.

$**p < 0.01$, $***p < 0.001$; unpaired student's t test. Error bar, mean ± SD, n = 3.

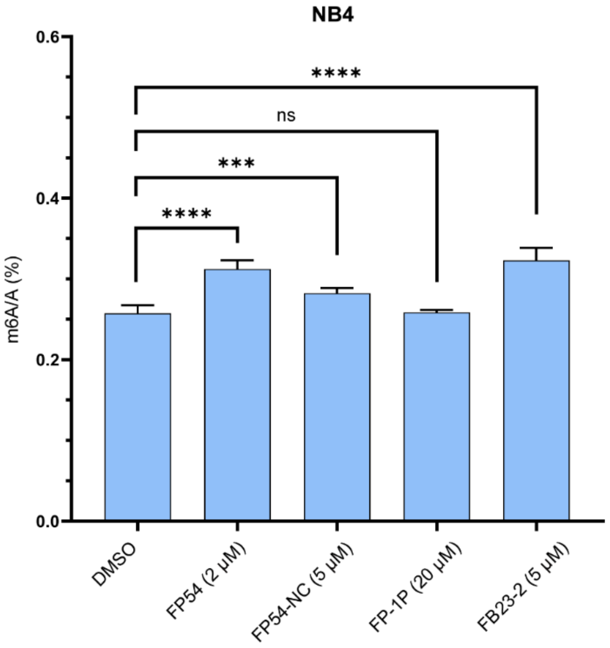

Figure S5. Quantitation of the percentage of m^6A/A ratios in mRNA by LC-MS/MS in NB4 cells treated with DMSO, FP54 (2 μM), FP54-NC (5 μM), FP-1P (20 μM) and FB23-2 (5 μM) for 48 h. ***$p < 0.001$, ****$p < 0.0001$; unpaired student's t test. Error bar, mean ± SD, n = 4.

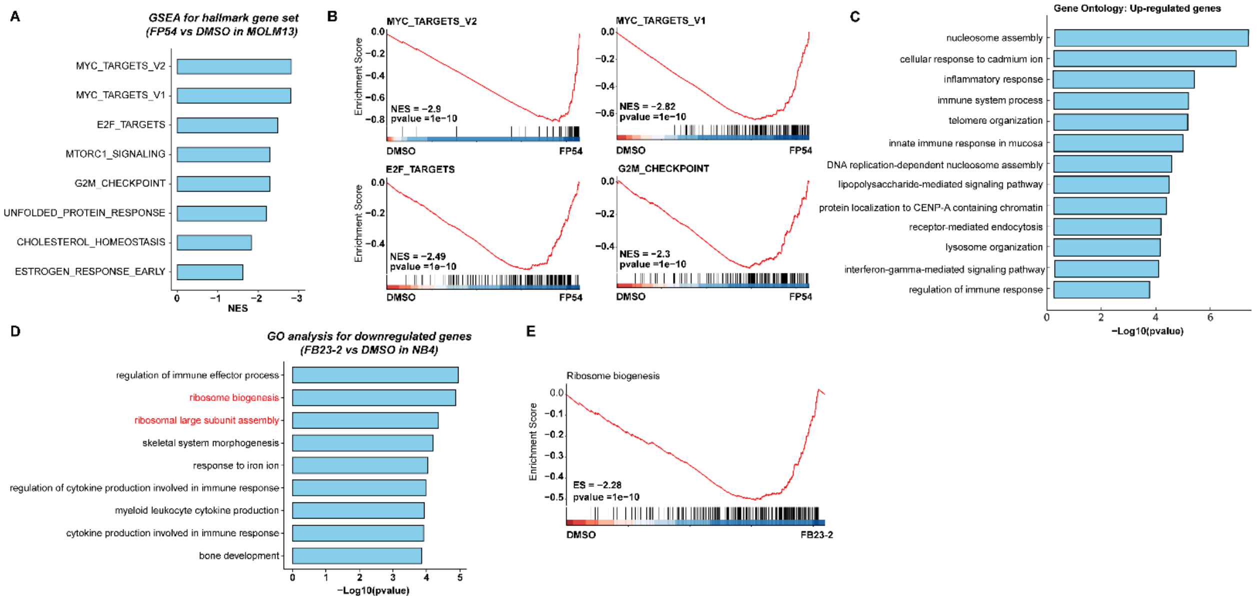

Figure S6. GSEA and GO analysis for genes in FP54- and FB32-2- treated AML cells.

(A) GSEA of downregulated genes after FP54 treatment in MOLM-13 cells.

(B) GSEA analysis for MYC targets, E2F targets and G2M targets after FP54 treatment in MOLM-13 cells.

(C) GO analysis for upregulated genes after FP54 treatment in MOLM-13 cells.

(D) Gene ontology analysis of differential genes after FB23-2 treatment in NB4 cells.

(E) GSEA analysis of ribosome biogenesis after FB23-2 treatment in NB4 cells.

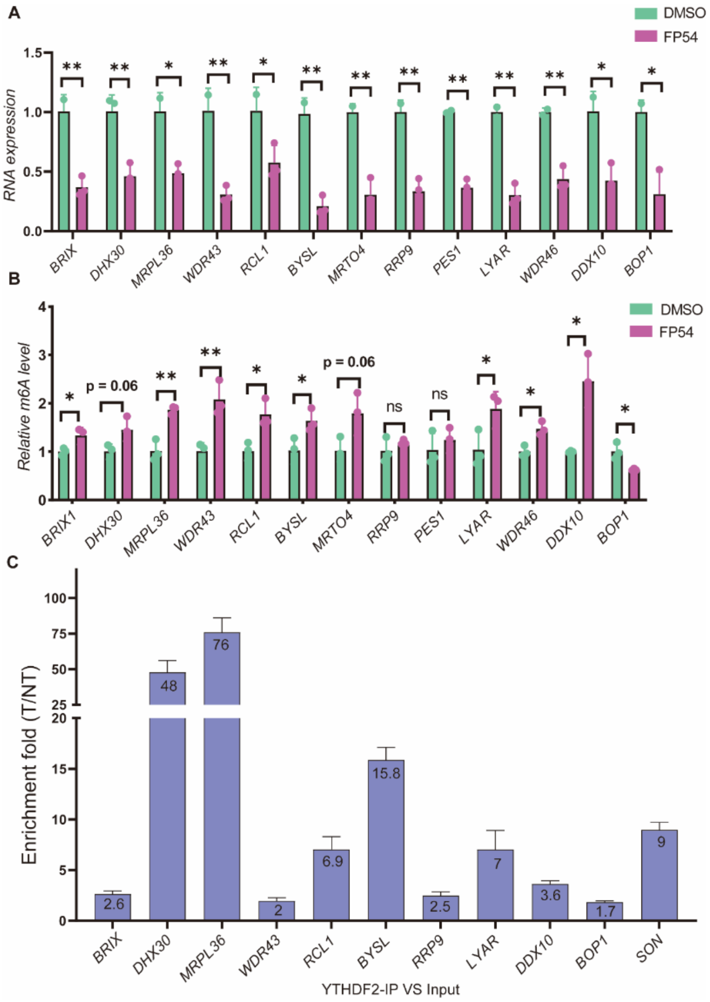

Figure S7. FTO degradation downregulates ribosome biogenesis-related mRNAs.

(A) RNA expressions of ribosome biogenesis-related mRNAs in MOLM-13 cells treated with 2 μM of FP54 for 48 h.

(B) Relative enrichment of ribosome biogenesis-related mRNAs in m^6A immunoprecipitation versus RNA input control.

(C) Relative enrichment folds of ribosome biogenesis-related mRNAs in YTHDF2-RNA coimmunoprecipitation versus RNA–protein input control. Data were normalized to the enrichment of non-target HPRT1 and calculated as fold enrichment relative to input.

**p < 0.01, ***p < 0.001; unpaired student's t test. Error bar, mean ± SD, n = 3, for C, n = 4.

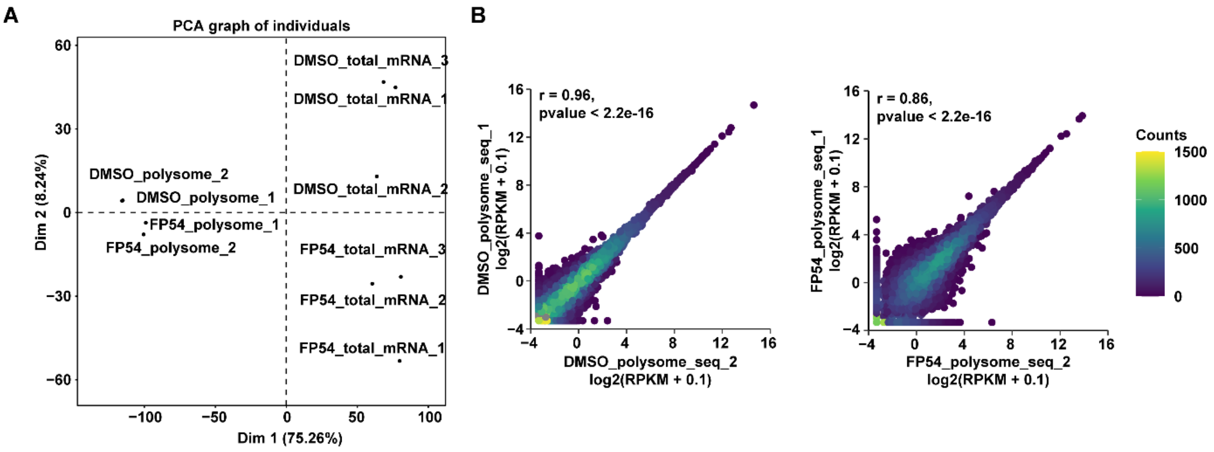

Figure S8. Quality control for Polysome-seq.

(A) Principle-component analysis (PCA) of both polysome-seq and RNA-seq data showed a clear separation between DMSO- and FP54-treated MOLM13.

(B) The linear regression curve demonstrated high reproducibility of polysome-seq results.

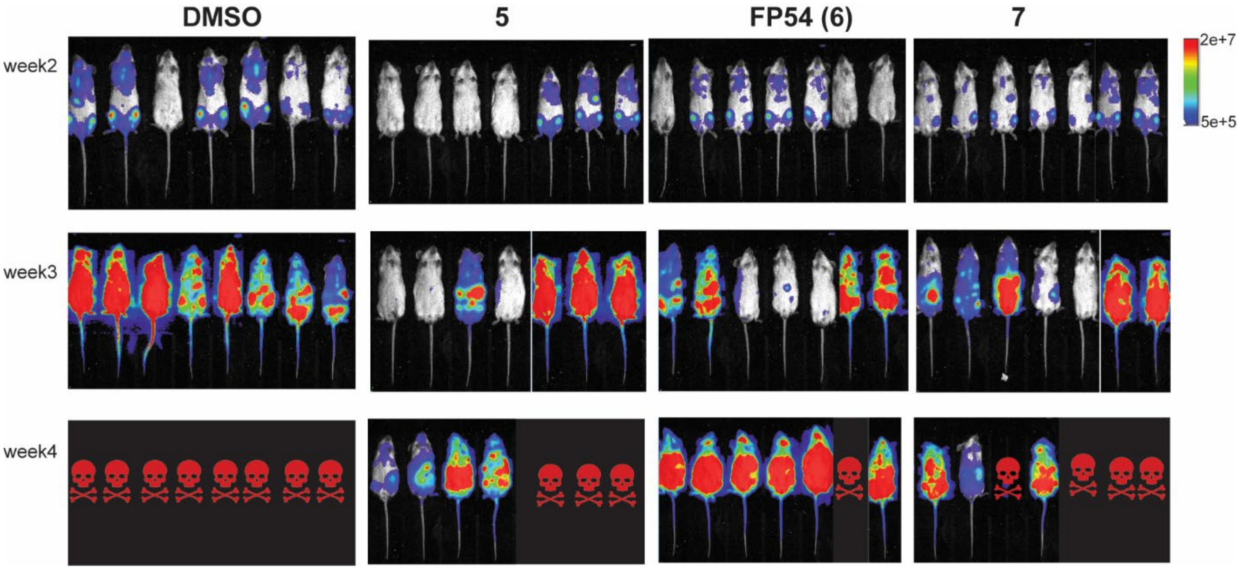

Figure S9. *In vivo* bioluminescence imaging of xenotransplantation mouse models with AML cells (MA9.3Ras) expressing luciferase after treatments with DMSO, **5**, **6** (FP54) and **7**.

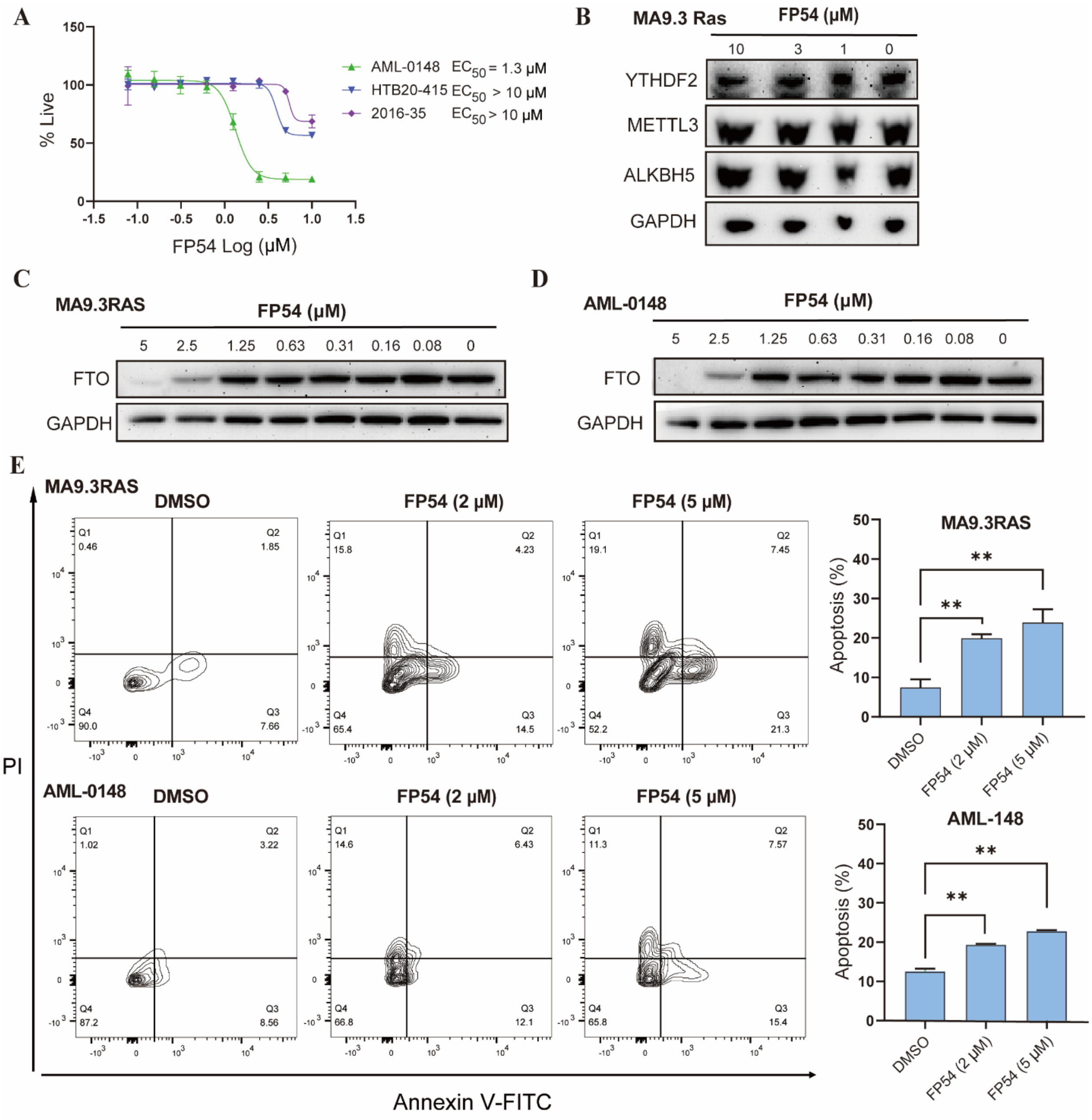

Figure S10. The cellular activity of FP54 in MA9.3RAS and PDX cells.

(A) The anti-proliferative activity of FP54 in three PDX AML cells. Plots shown as mean ± SD from n = 3 independent experiments.

(B) Immunoblots for YTHDF2, METTL3, ALKBH5 and GAPDH in MA9.3 Ras cells after treatment with FP54 at indicated concentrations for 48 h.

(C) Immunoblots for FTO in MA9.3RAS cells treated with FP54 at indicated concentrations for 48 h.

(D) Immunoblots for FTO in AML-0148 cells treated with FP54 at indicated concentrations for 48 h.

(E) Apoptosis quantification in MA9.3RAS and AML-0148 cells treated with DMSO and FP54 at indicated concentrations for 48 h.

**p < 0.01, unpaired student's t test. Error bar, mean ± SD, n = 3; Blots in B-D are representative of three independent experiments.

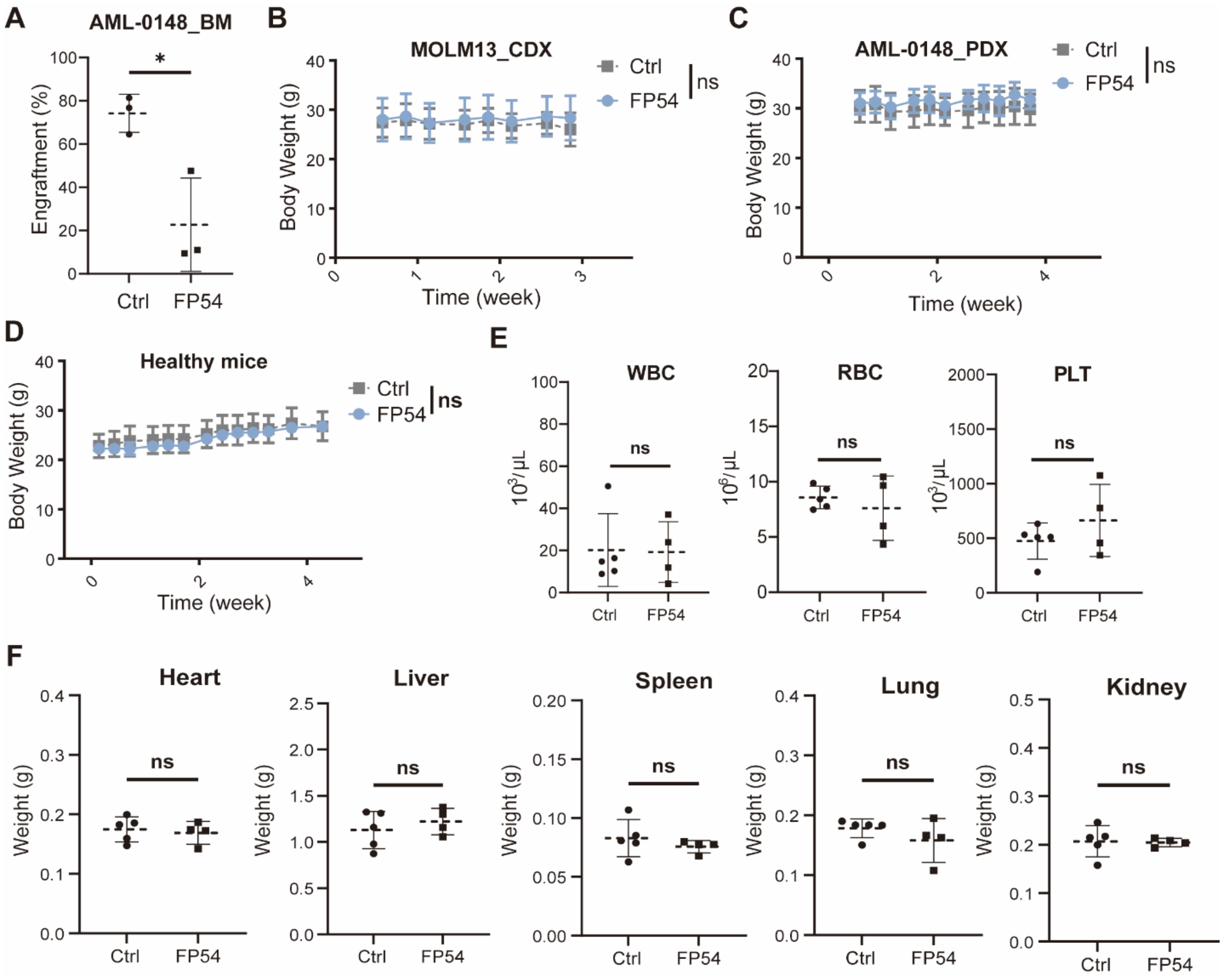

Figure S11. Toxicity evaluation of FP54 in mice.

(A) Statistical analysis of flow cytometry data from bone marrow (BM) of AML-0148 PDX mice collected at the disease endpoint for each animal across different treatment groups.

(B) Statistical analysis of body weight in MOLM13 CDX model during drug treatment.

(C) Statistical analysis of body weight in AML-0148 PDX model during drug treatment.

(D) Statistical analysis of body weight in healthy mice during drug treatment.

(E) Complete blood count analysis of peripheral blood in mice treated with FP54. White blood cell (WBC), red blood cell (RBC), and platelet (PLT) counts were measured in control and FP54-treated groups at the study endpoint. Data are presented as mean ± SD.

(F) Analysis of organ weights in control and FP54-treated mice. The weights of organs (heart, liver, spleen, lung and kidney) were measured at the study endpoint. Data are presented as mean ± SD.

For B, C and D, significance was determined using two-way ANOVA followed by Tukey's multiple comparisons test. $p < 0.05$ was considered statistically significant. For E and F, statistical significance was assessed using an unpaired two-tailed Student's *t*-test. $p < 0.05$ was considered statistically significant.

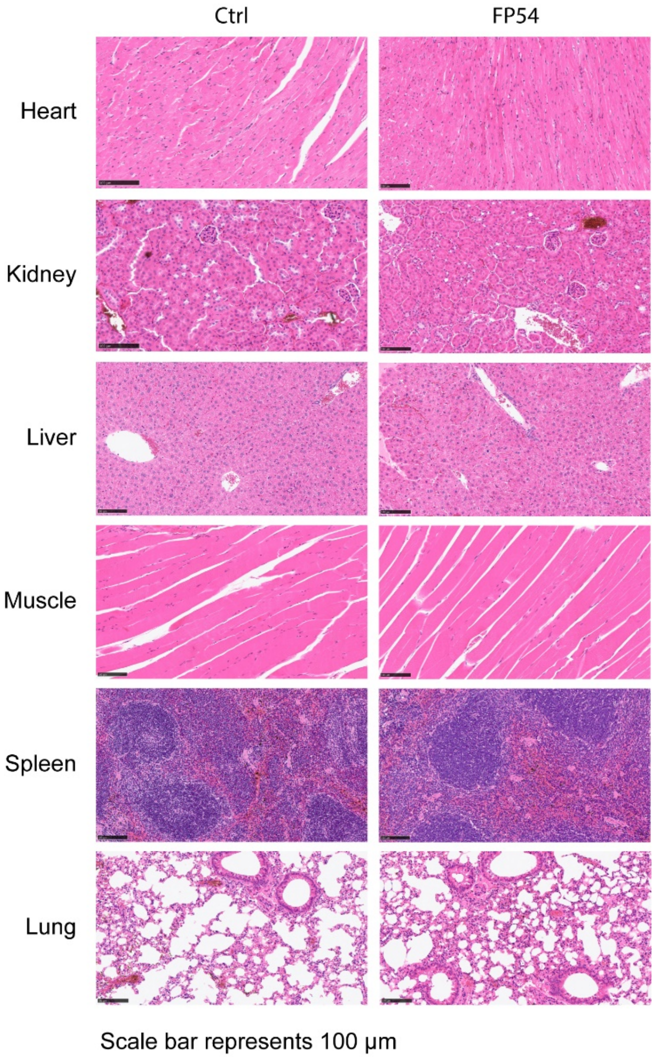

Figure S12. Representative Hematoxylin and Eosin (H&E) staining images of major organs from healthy mice treated with vehicle or FP54 for 14 days. Shown are sections of the heart, kidney, liver, muscle, spleen, and lung. Images are representative of at least three biological replicates per group (n ≥ 3). Scale bar, 100 μm.

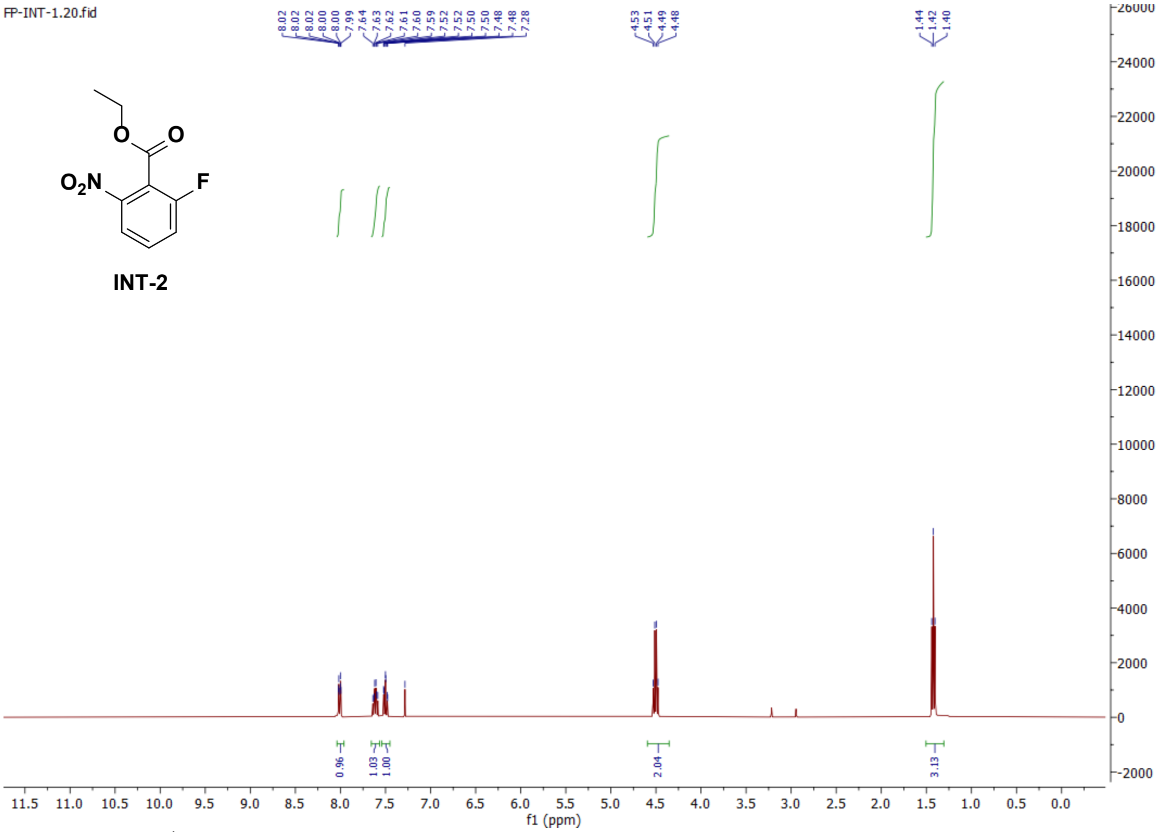

Figure S13. ^{1}H NMR spectrum of **INT-2**

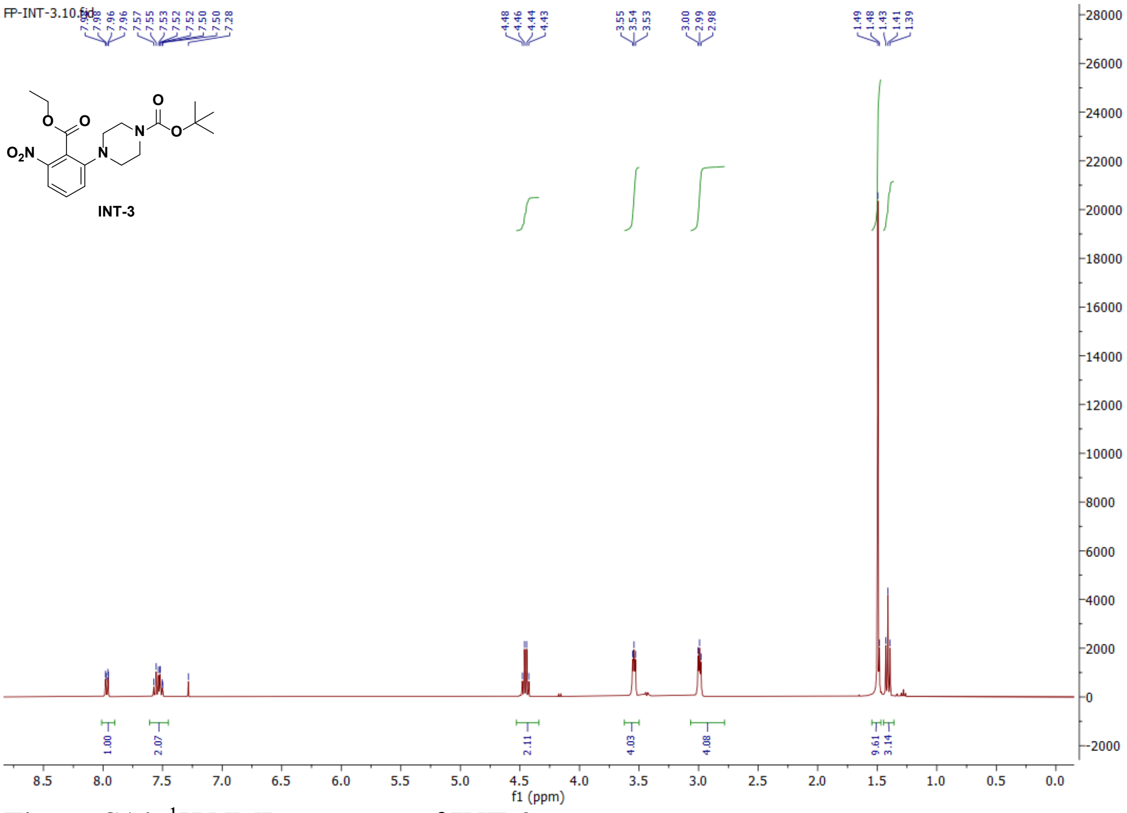

Figure S14. ^{1}H NMR spectrum of **INT-3**

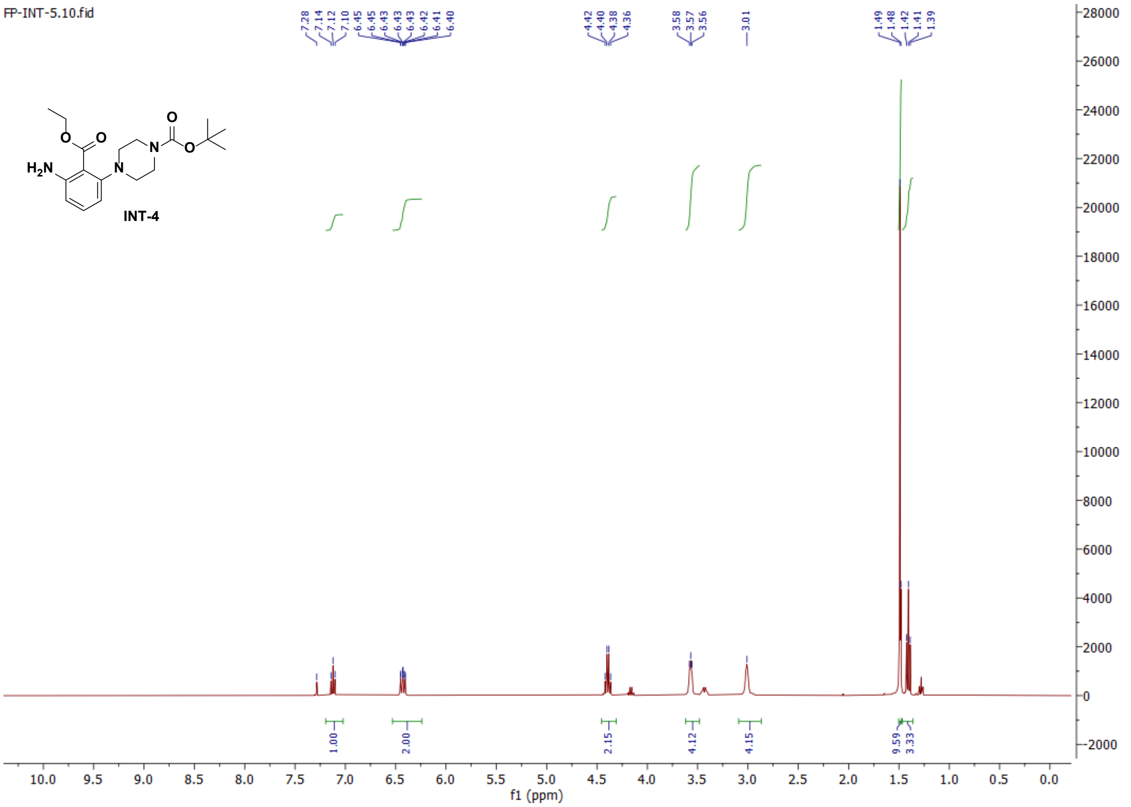

Figure S15. ^{1}H NMR spectrum of **INT-4**

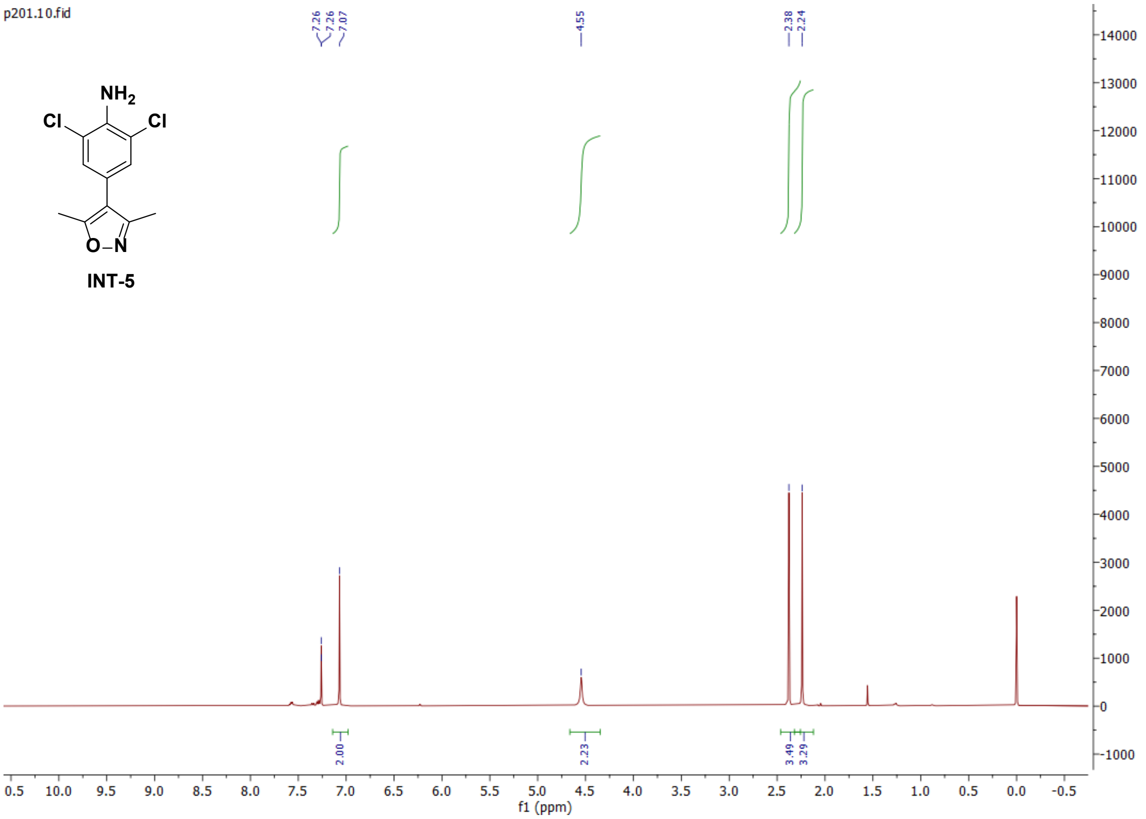

Figure S16. ^{1}H NMR spectrum of **INT-5**

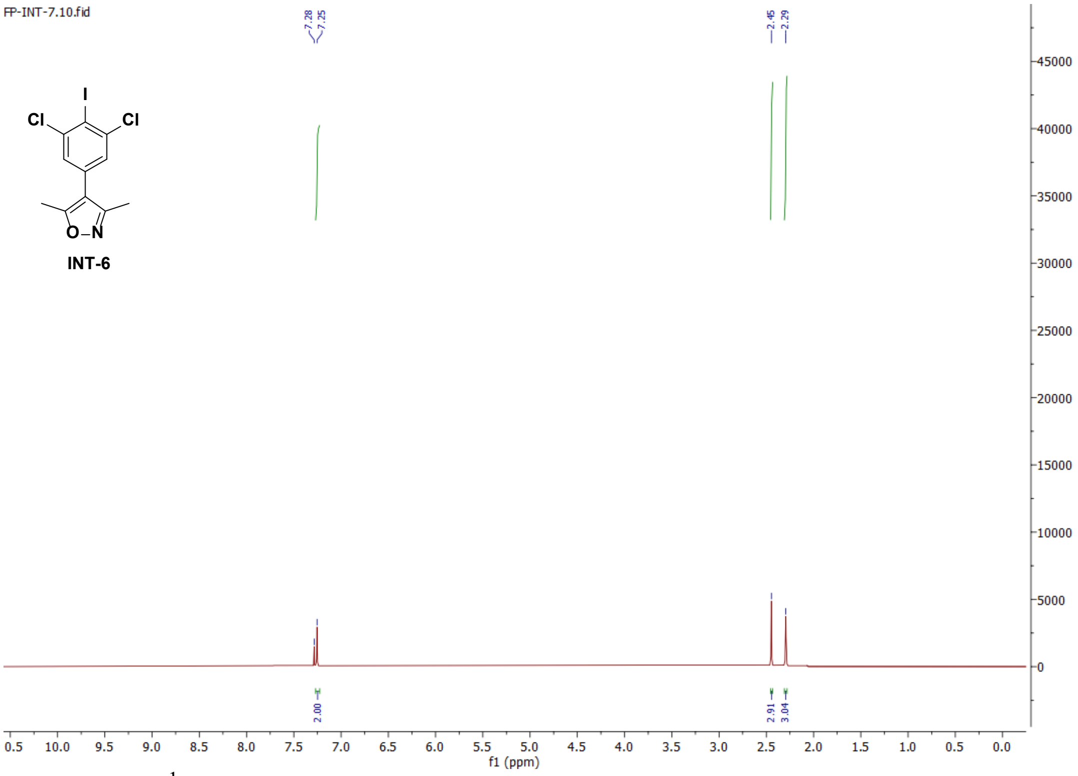

Figure S17. ^{1}H NMR spectrum of **INT-6**

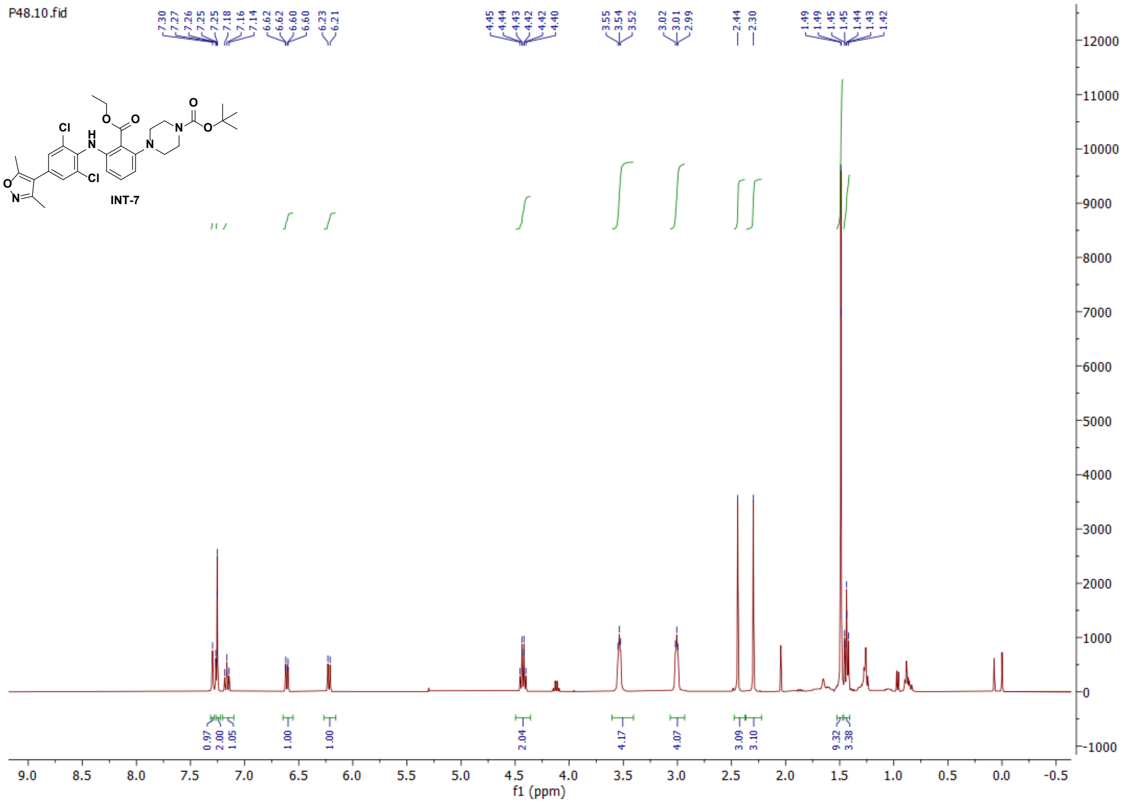

Figure S18. ^{1}H NMR spectrum of **INT-7**

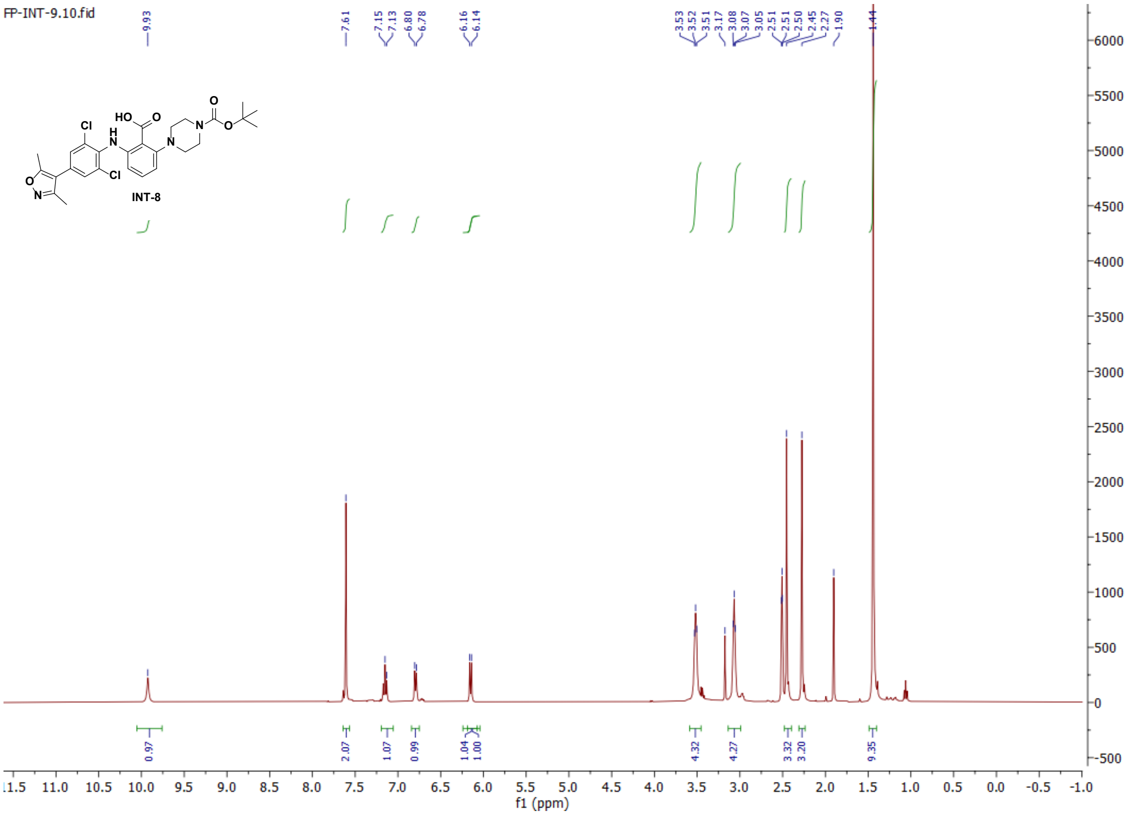

Figure S19. ^{1}H NMR spectrum of **INT-8**

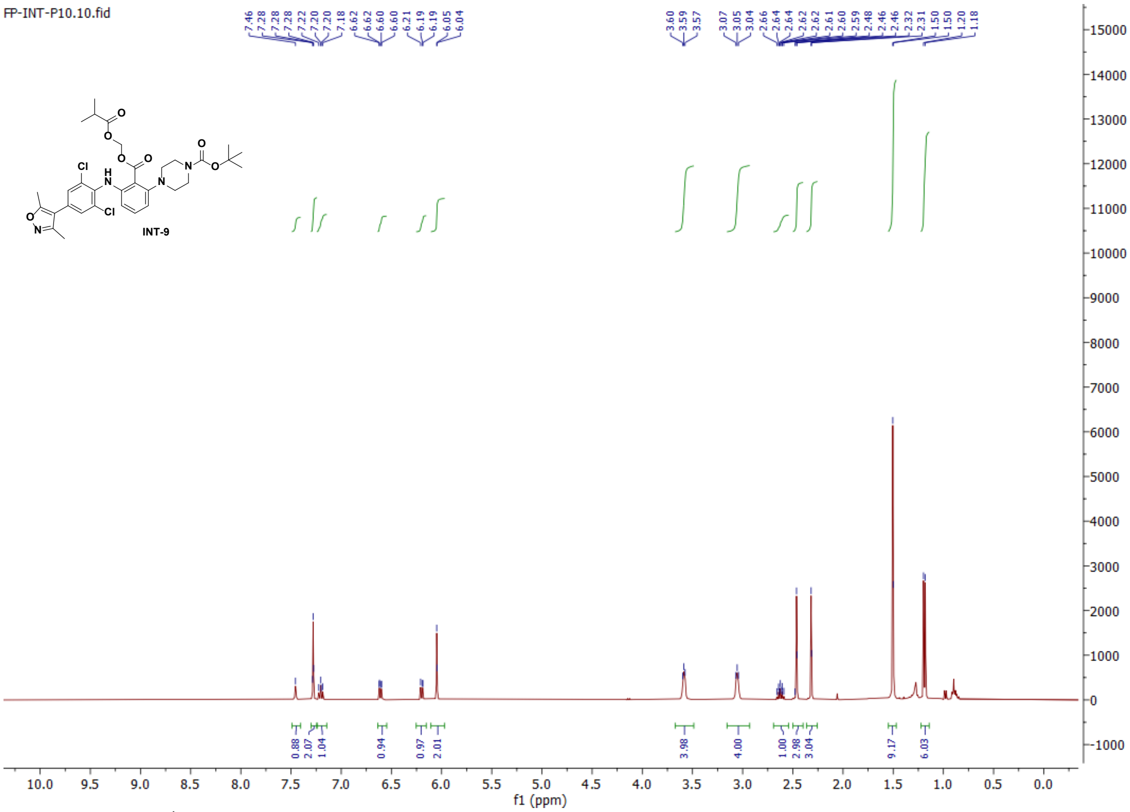

Figure S20. ^{1}H NMR spectrum of **INT-9**

A

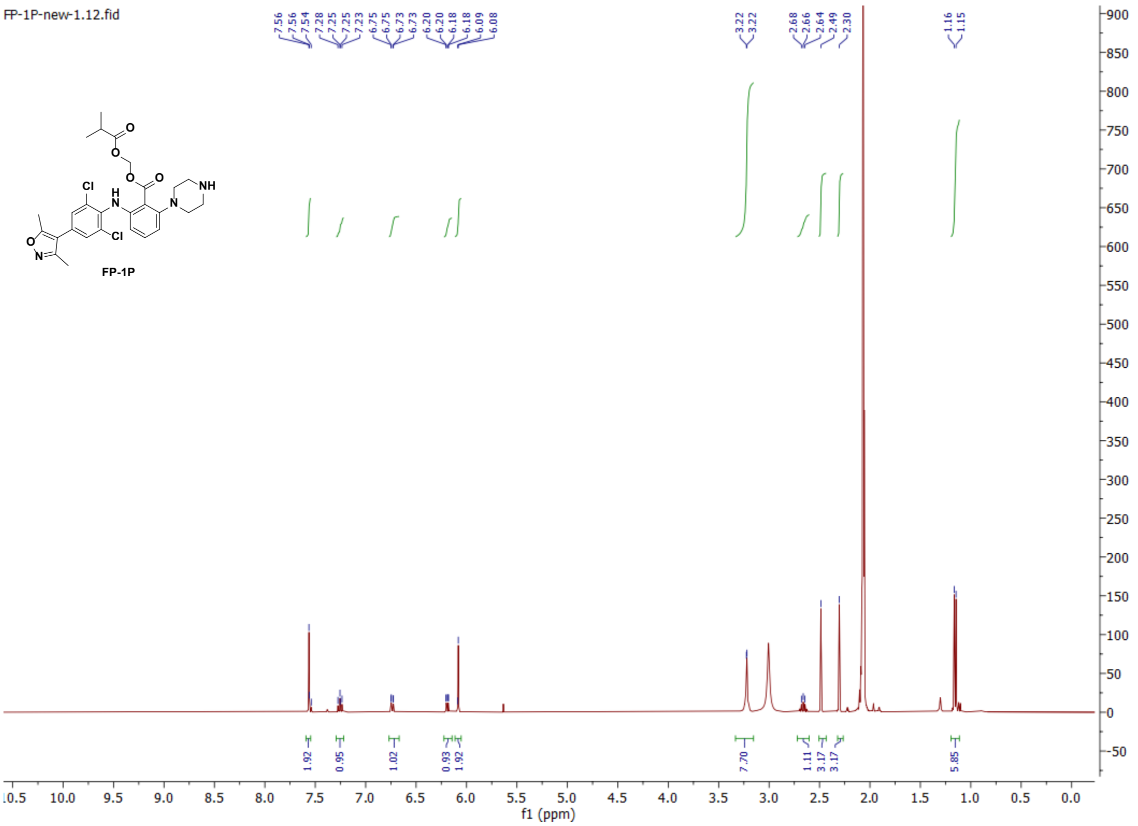

B

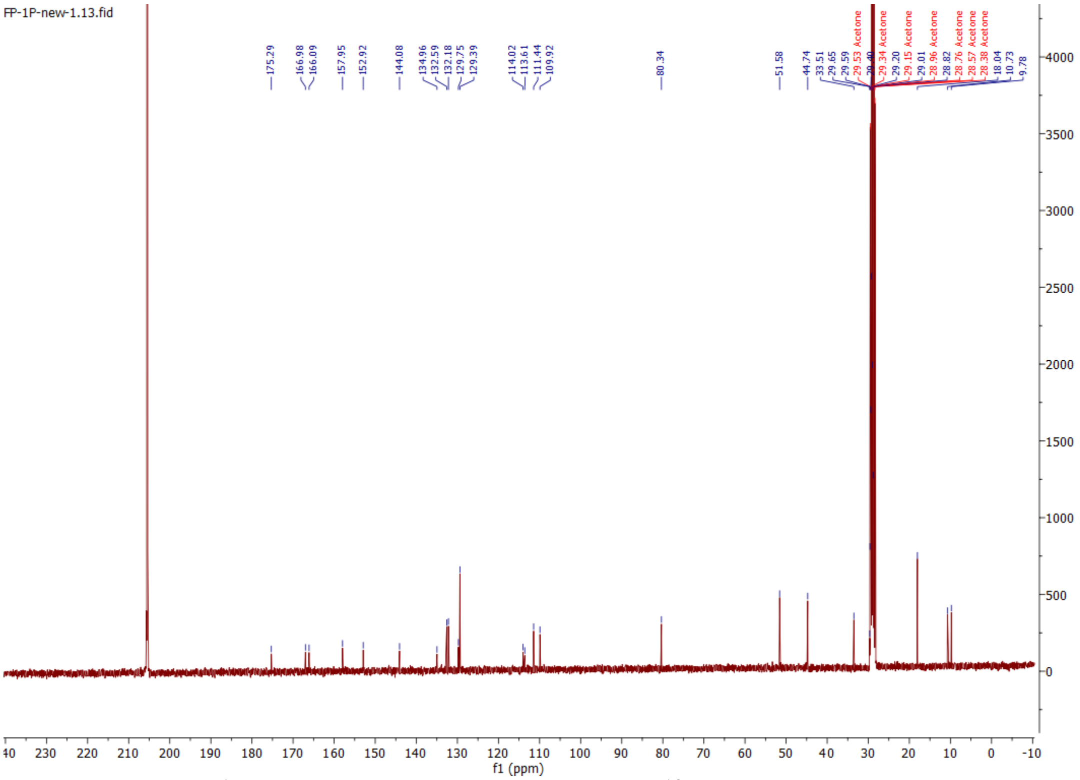

Figure S21. (A) ^{1}H NMR spectrum of **FP-1P**. (B) ^{13}C NMR spectrum of **FP-1P**.

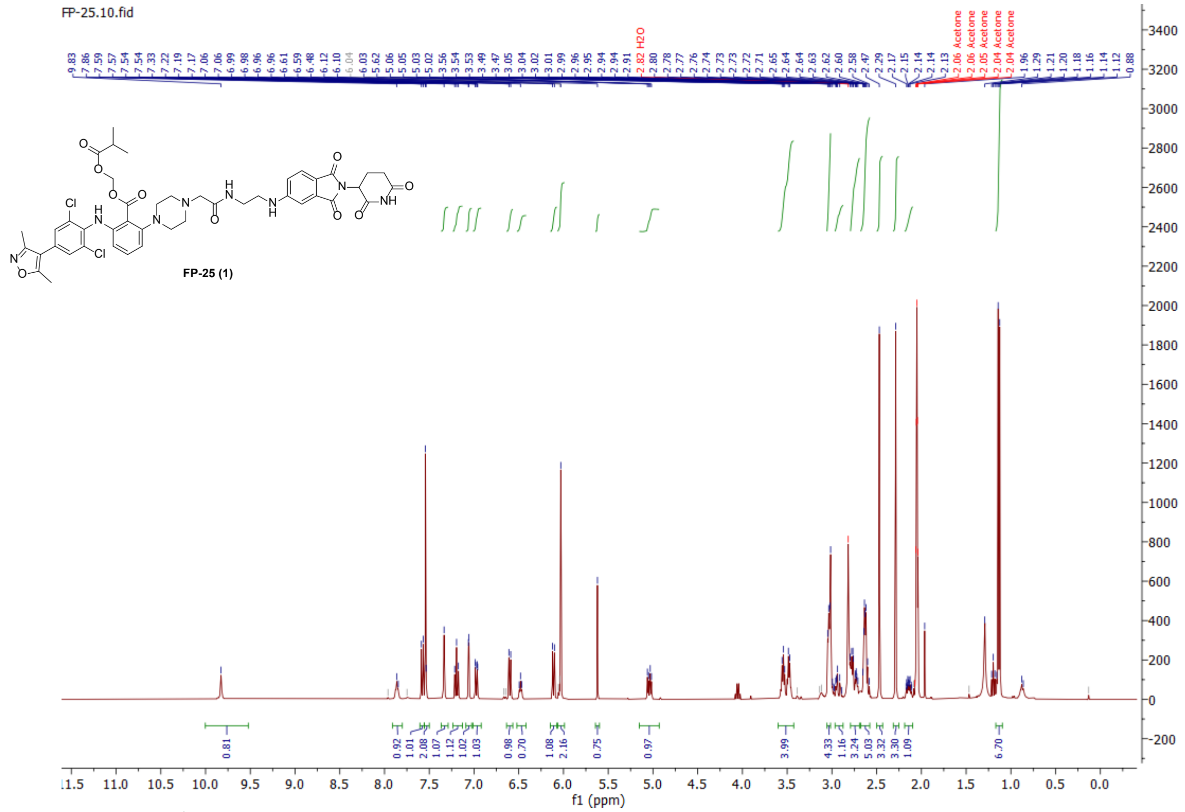

Figure S22. ^{1}H NMR spectrum of compound **1**

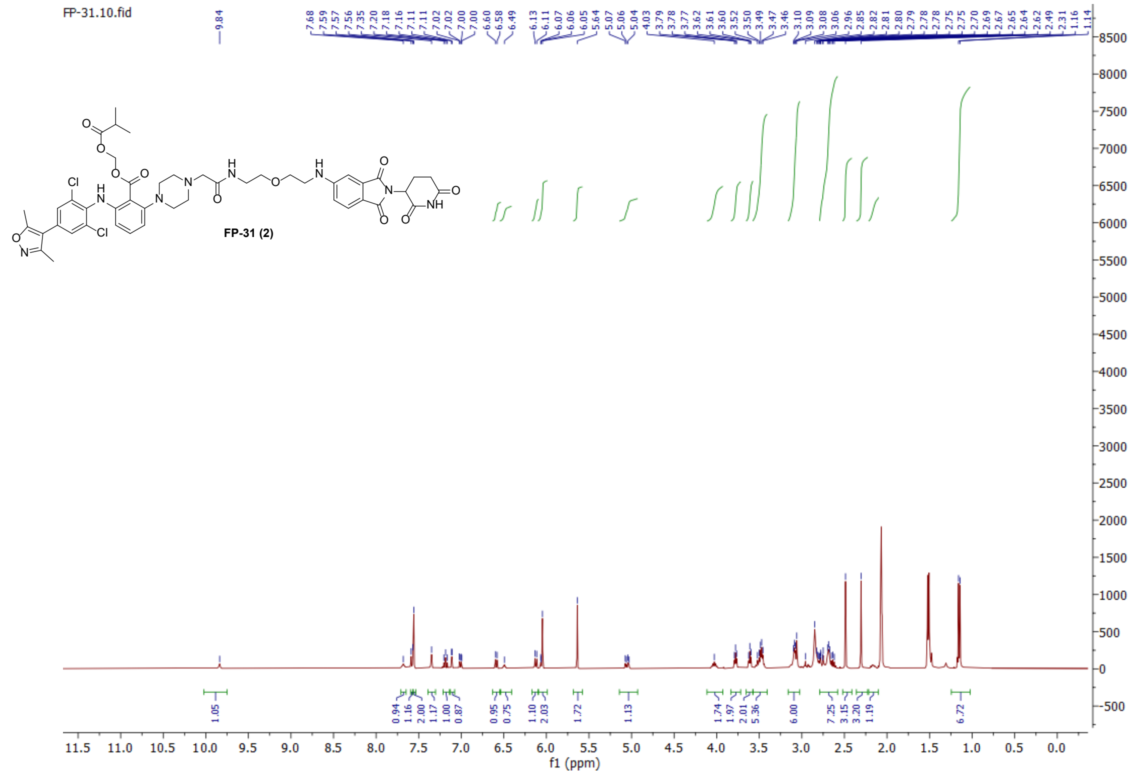

Figure S23. ^{1}H NMR spectrum of compound **2**

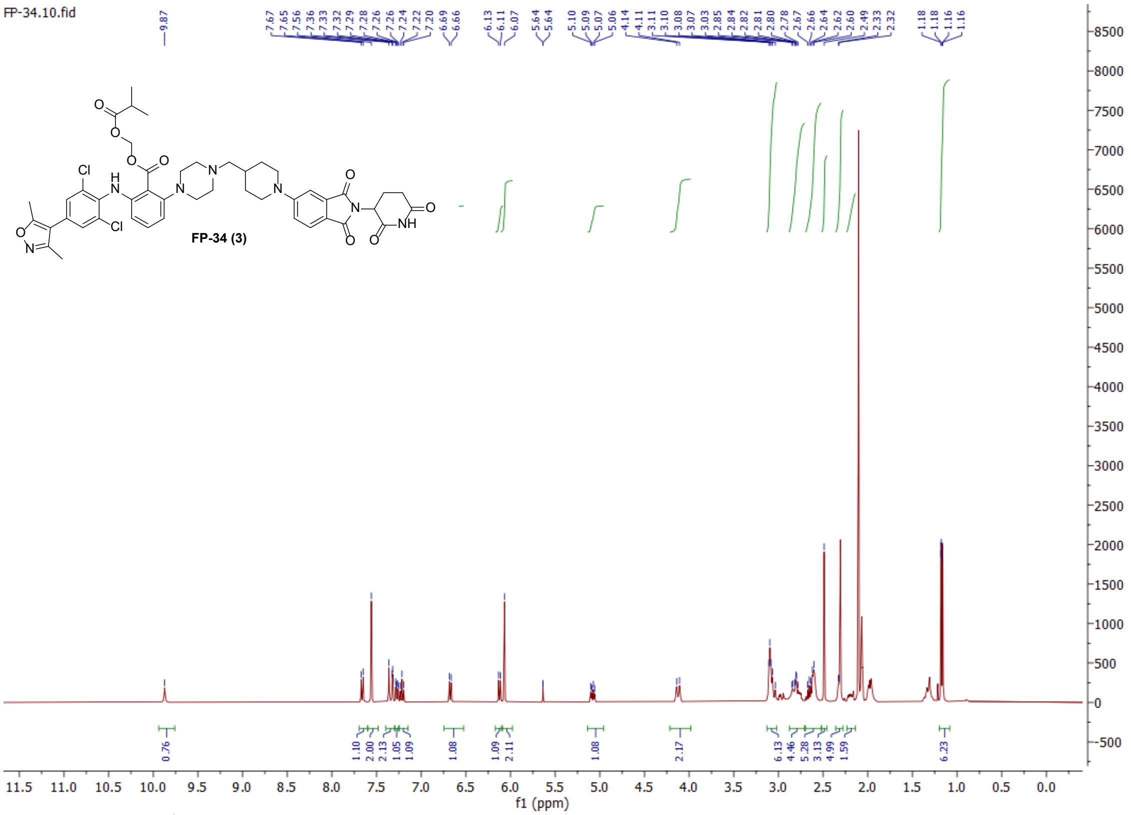

Figure S24. ^{1}H NMR spectrum of compound **3**

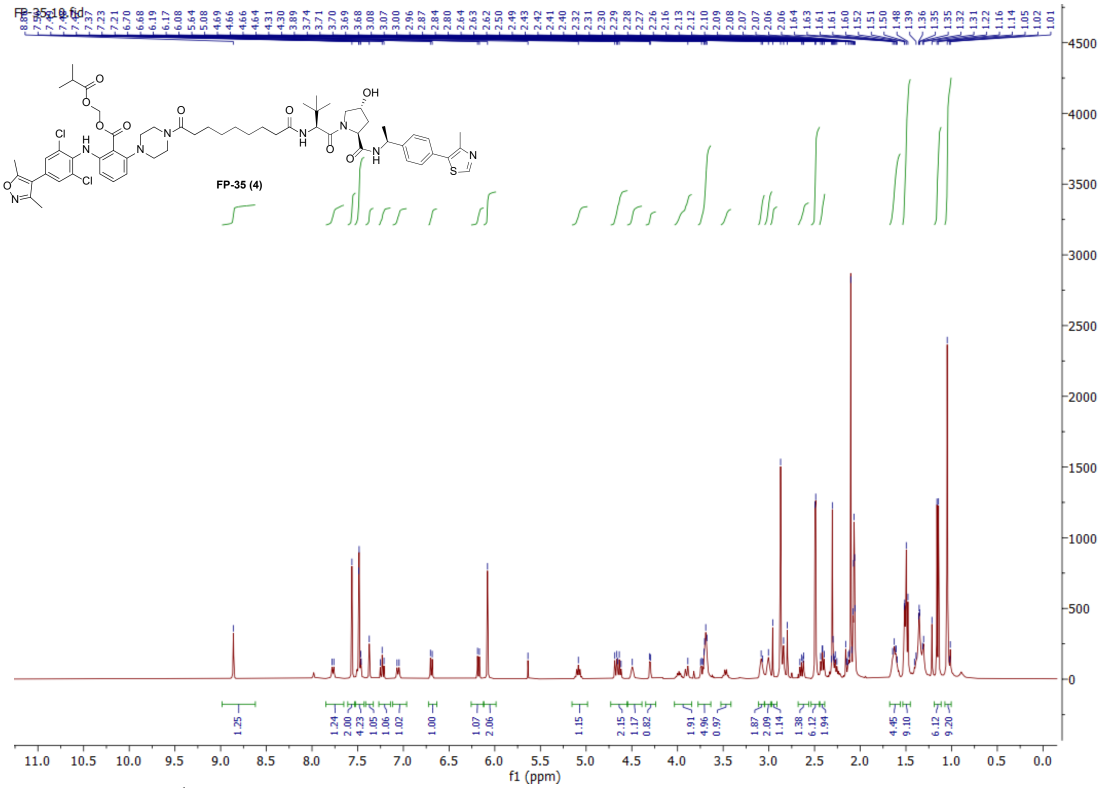

Figure S25. ^{1}H NMR spectrum of compound **4**

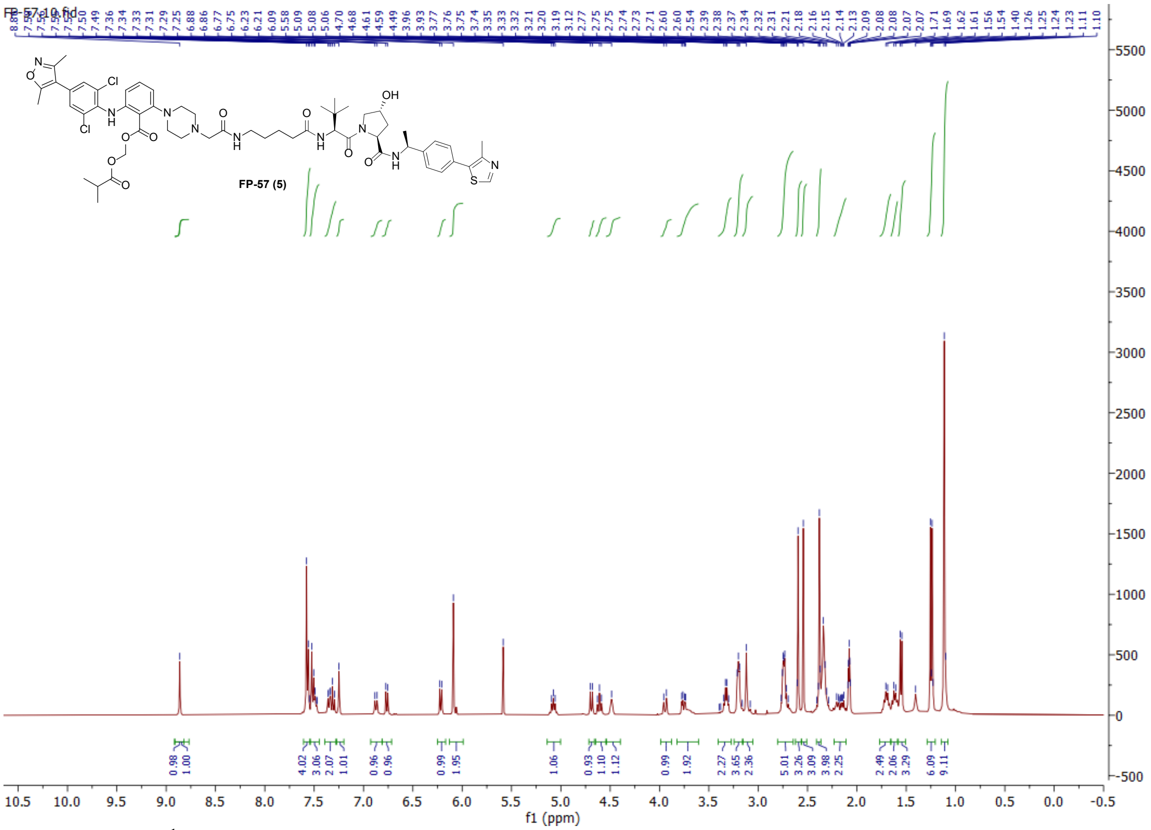

Figure S26. ^{1}H NMR spectrum of compound **5**

A

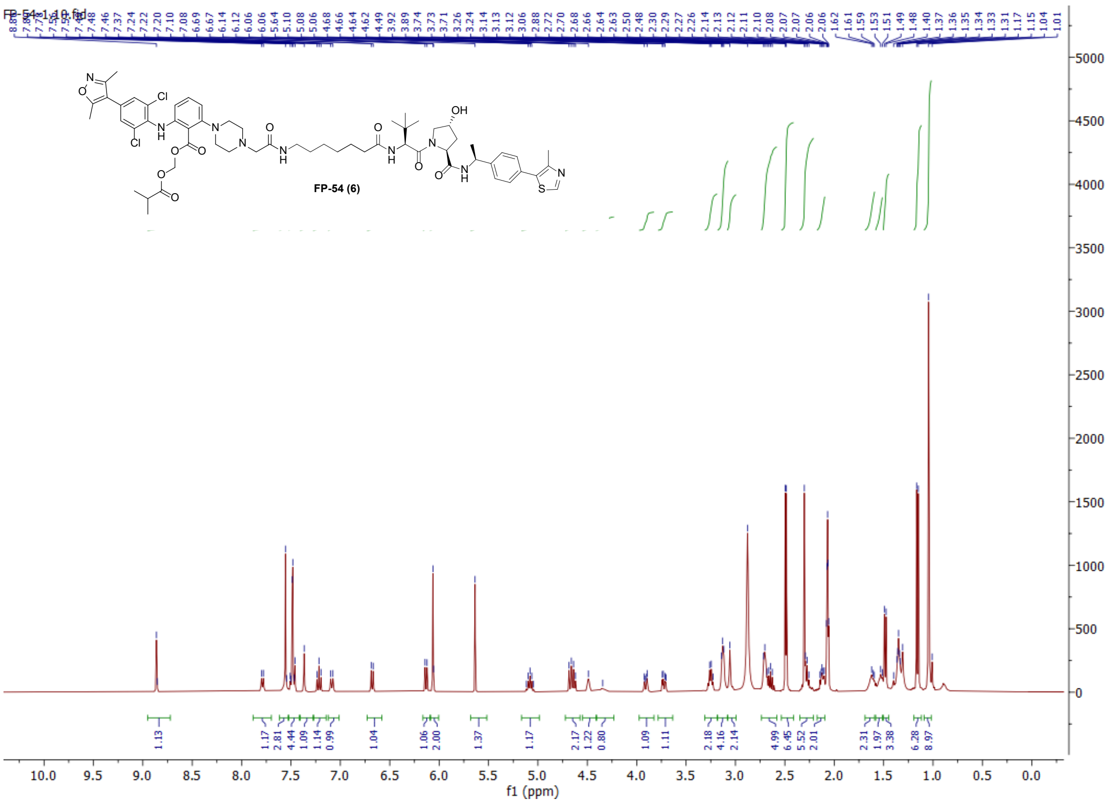

B

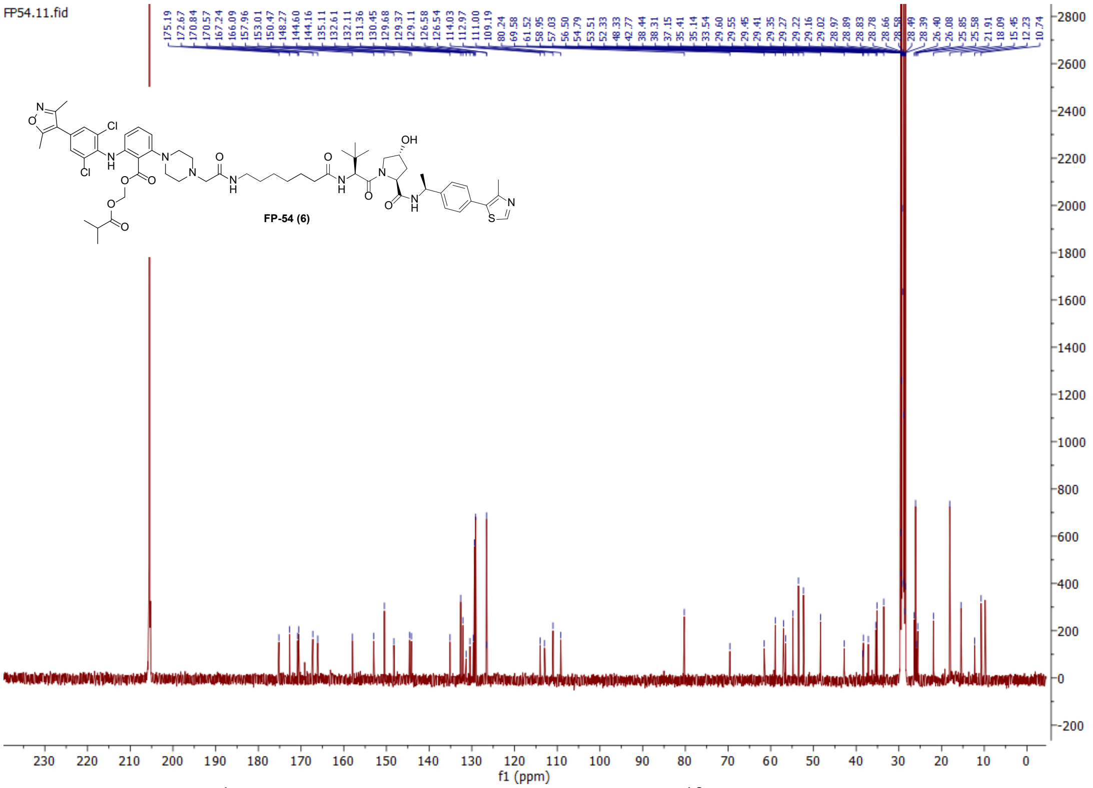

Figure S27. (A) ^{1}H NMR spectrum of FP54 (**6**); (B) ^{13}C NMR spectrum of FP54 (**6**)

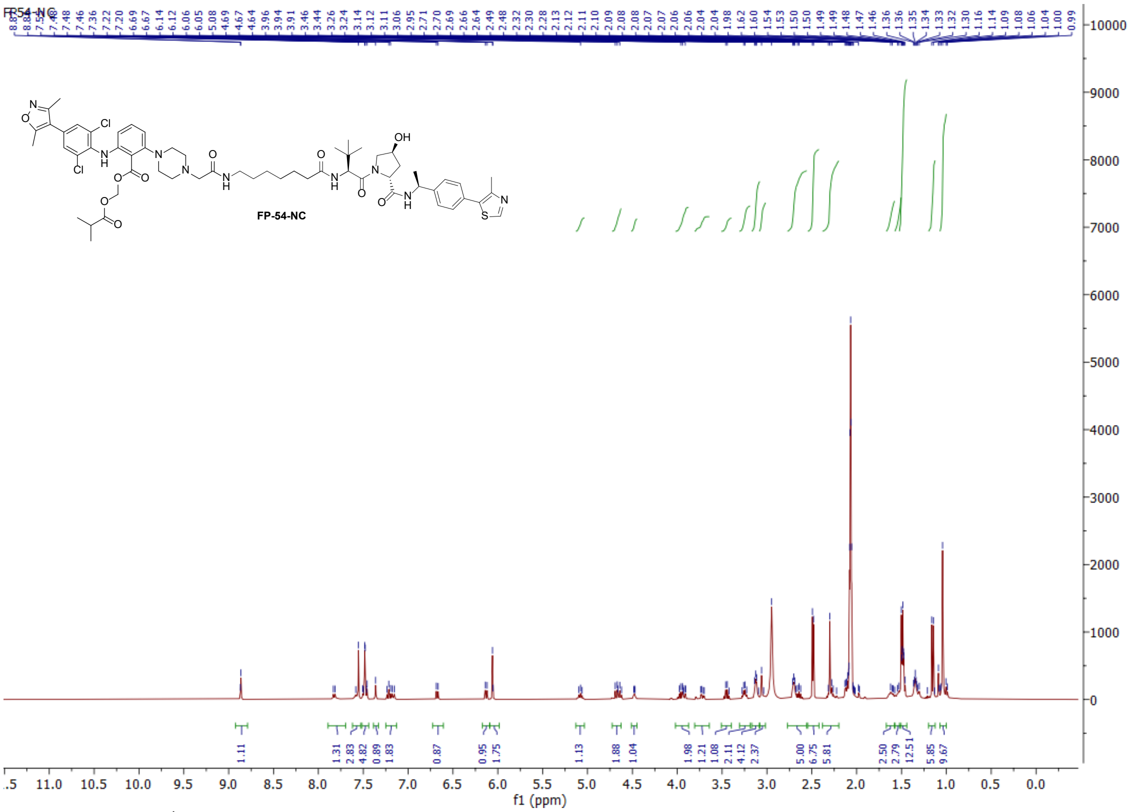

Figure S28. ^{1}H NMR spectrum of FP54-NC

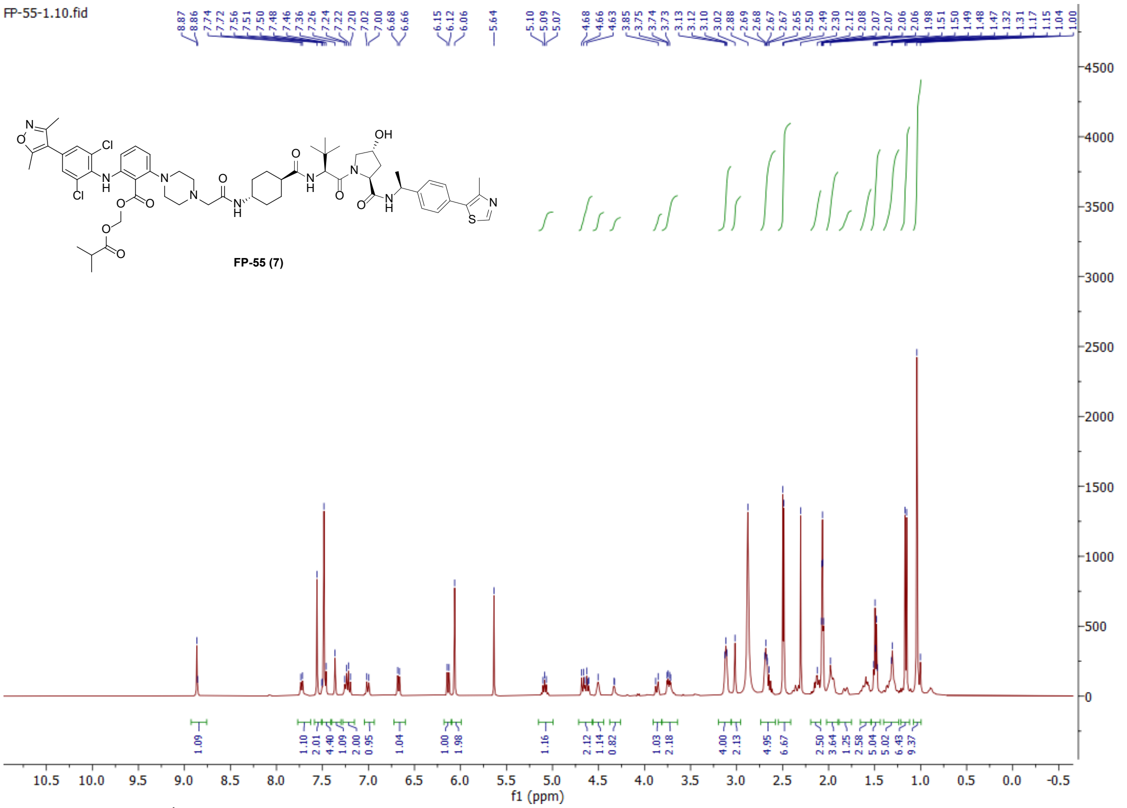

Figure S29. ^{1}H NMR spectrum of compound **7**

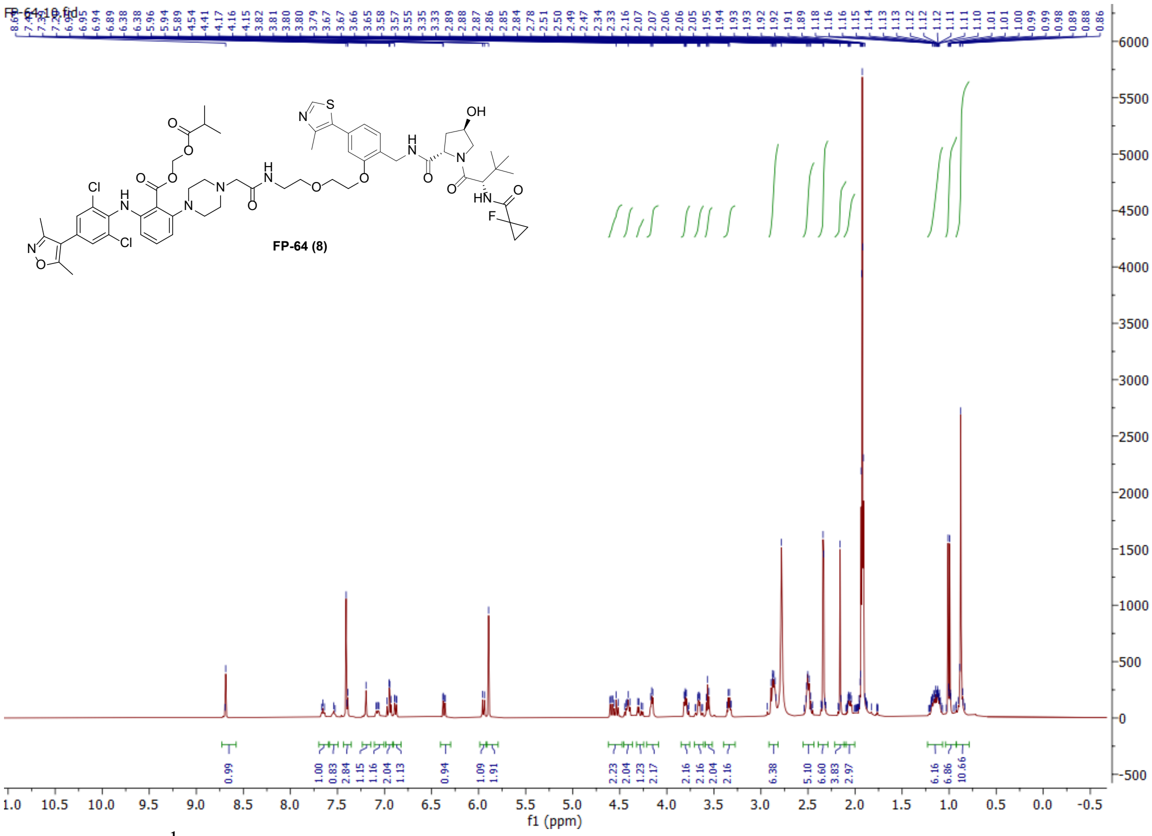

Figure S30. ^{1}H NMR spectrum of compound **8**

Name	Sequence
Brix-qPCR-F	CCAACCATTTGTGGACCACGTG
Brix-qPCR-R	TGGTCCTCCAAAACTTCCCTGG
DHX30-qPCR-F	GAGCACTACCTAGAGGACATCC
DHX30-qPCR-R	CGATGTGCAGAACCAGATCAGTC
MRPL36-qPCR-F	AGCAGTGCGCTCACTTCTCTCA
MRPL36-qPCR-R	CCTCTTCACCAGGTAACAGTCC
WDR43-qPCR-F	CCTCCACAAACCGAGCAAGTAG
WDR43-qPCR-R	GCTATTCGTCTGGAGGTCTTCC
RCL1-qPCR-F	CCTGTGAGGAAGGTCTTGAAGC
RCL1-qPCR-R	GTTCGCCATCTGAGGTGACACA
BYSL-qPCR-F	CTCTCCAACTGGGAGCAAATCC
BYSL-qPCR-R	TGCGTTCCTTCAGGTTAGAGGC
MRTO4-qPCR-F	ACAGCAAGCTGAAGGACATCCG
MRTO4-qPCR-R	GACCTGGTGCAGGTTGTCTTTG
RRP9-qPCR-F	GTTGGTGGCAAAAGAGATCCAGG
RRP9-qPCR-R	CTTTGGCAGCAGAGAAGATGGC
PES1-qPCR-F	TCGTGTGGATCACTCCCTATGC
PES1-qPCR-R	GGTTGAGCAACTGGTAAAGGCG
LYAR-qPCR-F	CCATTCTGGACCAGGTGTGGAA
LYAR-qPCR-R	ACTTTGGAGGCTGGAACCTTGG
WDR46-qPCR-F	ACCTACCTGGATGTGTCAGTGG
WDR46-qPCR-R	AGTGGCTCCTTCATAGCTGGAC
DDX10-qPCR-F	GAGTTTGTCCGTAAGAGAGCTGC
DDX10-qPCR-R	GGCAGTTCTACCTGCTCTGTGA
BOP1-qPCR-F	AATACCTGCTCAGCGAGGAGGA
BOP1-qPCR-R	GTTCCTGGATGAAGCGTCCGTA
AURKA-qPCR-F	GCAACCAGTGTACCTCATCCTG
AURKA-qPCR-R	AAGTCTTCCAAAGCCCACTGCC
STAG1-qPCR-F	GCAGCCTACTTGGTGGACAGTT
STAG1-qPCR-R	GAGCACTCTCTTGACGATCAGAC
GAPDH-qPCR-F	GTCTCCTCTGACTTCAACAGCG
GAPDH-qPCR-R	ACCACCCTGTTGCTGTAGCCAA
18S-qPCR-F	CGGACATCTAAGGGCATCAC
18S-qPCR-R	AACGAACGAGACTCTGGCAT

Table S1. List of primers used for this study. All primers were synthesized by Integrated DNA Technologies (IDT).

Name	Source	Identifier
FTO	Abcam	ab126605
ALKBH5	Abcam	ab195377
YTHDF2	ProteinTech Group INC	24744-1-AP
METTL3	ProteinTech Group INC	15073-1-AP
GAPDH	Cell Signaling Tech	2118S
STAG1	ProteinTech Group INC	14015-1-AP
AURKA	Cell Signaling Tech	14475T

Table S2. List of immunoblotting antibodies used for this study.

Patient ID	Sex	Age	Karyotype	Genetic alterations	Disease Status
HTB22-0148	N/A	N/A	Normal	DNMT3A mutation, IDH2 mutation	Newly Diagnosed
#20-415	Female	54	47,XX,+21[19];47,sl,del(9)(q?13q22)[3]	CEBPA Mutation (c.232del; p.Leu78Trpfs*82); CEBPA Mutations (c.913_921dup; p.Gln305_Asn307dup)"	Relapsed/Refractory
#2016-35	Female	2	Normal	KDM5C mutation	Relapsed/Refractory

Table S3. Information of the AML patient samples used in the study.

Table S4. Polysome-seq results from MOLM-13 cells treated with DMSO or FP54. Polysome profiling was performed on MOLM-13 cells treated with DMSO (vehicle) or 2 μM FP54 for 48 h. Translation efficiency (TE) was calculated as the ratio of normalized read counts in polysome-associated mRNA to total mRNA.
